# Tumor-Associated Microbiota in Esophageal Squamous Cell Carcinoma

**DOI:** 10.3389/fcell.2021.641270

**Published:** 2021-02-18

**Authors:** Weixiong Yang, Chang-Han Chen, Minghan Jia, Xiangbin Xing, Lu Gao, Hsin-Ting Tsai, Zhanfei Zhang, Zhenguo Liu, Bo Zeng, Sai-Ching Jim Yeung, Mong-Hong Lee, Chao Cheng

**Affiliations:** ^1^Department of Thoracic Surgery, The First Affiliated Hospital of Sun Yat-sen University, Guangzhou, China; ^2^Department of Applied Chemistry, and Graduate Institute of Biomedicine and Biomedical Technology, National Chi Nan University, Nantou County, Taiwan; ^3^Department of Breast Cancer, Cancer Center, Guangdong Provincial People’s Hospital, Guangdong Academy of Medical Sciences, Guangzhou, China; ^4^Department of Gastroenterology, The First Affiliated Hospital, Sun Yat-sen University, Guangzhou, China; ^5^BGI Genomics, BGI-Shenzhen, Shenzhen, China; ^6^Department of Emergency Medicine, The University of Texas MD Anderson Cancer Center, Houston, TX, United States; ^7^Guangdong Research Institute of Gastroenterology, The Sixth Affiliated Hospital of Sun Yat-sen University, Guangzhou, China; ^8^Guangdong Provincial Key Laboratory of Colorectal and Pelvic Floor Disease, The Sixth Affiliated Hospital of Sun Yat-sen University, Guangzhou, China

**Keywords:** esophageal squamous cell carcinoma, microbiota, microbial dysbiosis index, carcinogenesis, *Fusobacteria*

## Abstract

Important evidence indicates the microbiota plays a key role in esophageal squamous cell carcinoma (ESCC). The esophageal microbiota was prospectively investigated in 18 patients with ESCC and 11 patients with physiological normal (PN) esophagus by 16S rRNA gene profiling, using next-generation sequencing. The microbiota composition in tumor tissues of ESCC patients were significantly different from that of patients with PN tissues. The ESCC microbiota was characterized by reduced microbial diversity, by decreased abundance of *Bacteroidetes, Fusobacteria*, and *Spirochaetes*. Employing these taxa into a microbial dysbiosis index demonstrated that dysbiosis microbiota had good capacity to discriminate between ESCC and PN esophagus. Functional analysis characterized that ESCC microbiota had altered nitrate reductase and nitrite reductase functions compared with PN group. These results suggest that specific microbes and the microbiota may drive or mitigate ESCC carcinogenesis, and this study will facilitate assigning causal roles in ESCC development to certain microbes and microbiota.

## Introduction

Esophageal cancer is the ninth most common cancer and the sixth most common cause of cancer death worldwide (Pennathur et al., 2013; Bray et al., 2018). The two major subtypes of esophageal carcinoma are adenocarcinoma and esophageal squamous cell carcinoma (ESCC), with the latter accounting for the dominate histopathological type in the Chinese population (Abnet et al., 2018). These two subtypes are epidemiologically and biologically distinct. Despite reasonable progress in the diagnosis and treatment of ESCC, the prognosis of the patients remains very poor, with a 5-year survival rate of only about 15–25% (Pennathur et al., 2013; Rustgi and El-Serag, 2014). The pathogenesis of ESCC has not been well elucidated, which restricts the effective prevention and treatment of this disease. The incidence of esophageal carcinoma remains a health care challenge worldwide. For ESCC, there are very few targeted therapies available and survival rates remains dismal. Therefore, there is an urgent need to characterize biomarkers/therapy strategy for esophageal carcinoma. Further studies are needed to clarify the pathogenesis of esophageal cancer and to explore new diagnostic and therapeutic possibilities.

The intestinal tract microbiota, containing at least 38 trillion bacteria, is critical for the maintenance of homeostasis and health (Fischbach, 2018). Progress in metagenome-wide association studies of fecal samples has characterized some important microbial markers of CRC (Schwabe and Jobin, 2013; Zou et al., 2018), and the causal effect of bacteria on cancer has been recognized (Yu et al., 2017). Also, the microbiome has been discovered to be involved in the initiation and progression of various types of cancer, such as liver cancer (Schwabe and Jobin, 2013; Louis et al., 2014; Ma et al., 2018). Experimental evidence indicates that the human intestinal microbiome can influence tumor development and progression in the gastrointestinal tract by damaging DNA, activating oncogenic signaling pathways, producing tumor-promoting metabolites, and suppressing the antitumor immune response (Nougayrede et al., 2006; Kostic et al., 2013; Rubinstein et al., 2013; Schwabe and Jobin, 2013; Ridlon et al., 2016; Zou et al., 2018). Esophagus is an important part of the upper digestive system and more and more attentions are paid to the relationship between microbiome and esophageal cancer. Esophageal cancer is one of the most aggressive malignant cancers. Treatment strategies provided by conventional therapies have limited improvements in clinical outcome. It is then critical to seek innovative clinical strategies for treating this type of cancer. As intestinal tract microbiota plays important roles during tumorigenesis, exploiting microbiota for cancer prevention or treatment may be feasible.

However, only a small number of studies characterized the human esophageal microbiota in health and disease (Baba et al., 2017). The major findings in esophageal adenocarcinoma were that lipopolysaccharides, a major structure of the outer membrane in gram-negative bacteria, can upregulate gene expression of proinflammatory cytokines via activation of the Toll-like receptor 4 and NF-κB pathway and promote the occurrence of Barrett esophagus and adenocarcinoma (Yang et al., 2012; Zhang et al., 2013). Host interactions with microbiota in esophagitis, Barrett’s esophagus, esophageal adenocarcinoma and ESCC can be different. Here we focused on the microbiota of ESCC. As for ESCC, the microbiome was less well characterized. Only few studies suggested that the change of microbiota such as Fusobacterium were associated with the occurrence of ESCC (Yu et al., 2014; Shao et al., 2019). The association between the change of esophageal microbiome and ESCC development has not been well elucidated (Di Pilato et al., 2016; Baba et al., 2017). Therefore, to investigate the relationship between the changes in esophageal mucosal microbiota and the occurrence of ESCC, we conducted a prospective study and performed high-throughput profiling of the esophageal mucosal microbiota in ESCC cases and normal controls. The next-generation sequencing (NGS) of the 16S rRNA gene were used to determine microbiota communities potentially associated with ESCC. We have demonstrated microbial relative abundances at the phylum and genus level for ESCC. We illustrated the impact of ESCC-associated bacterial taxa during the pathogenesis of ESCC.

## Materials and Methods

### Patients

This prospective observational study was conducted according to a protocol approved by the respective Institutional Ethics Committees of the First Affiliated Hospital of Sun Yat-sen University and according to the Declaration of Helsinki. This GASTO1039 study was registered at http://www.chictr.org.cn/(the Chinese Clinical Trial Registry: ChiCTR1800018897). Eighteen patients with esophageal squamous cell carcinoma (ESCC) and eleven volunteers physiological normal (PN) esophagus from the First Affiliated Hospital of Sun Yat-sen University were included in the discovery cohort from July 2017 to March 2018. A validation cohort of an additional 20 ESCC patients and 4 volunteers were enrolled from April 2018 to December 2018 (Supplementary Tables 1, 2). The patients who took the medicine such as antibiotics, hormones, intestinal flora regulator, proton pump inhibitors, etc or had clinical infection were excluded. Tissues for analysis came from patients undergoing routine esophagogastroduodenoscopy for the investigation of upper gastrointestinal symptoms or surgical resection. All patients provided written informed consents.

### 16S rDNA Gene Sequencing

DNA was extracted from healthy group and tumor group according to the protocol of E.Z.N.A. Bacterial DNA Kit (Omega Bio-tek, Norcross, GA, United States). The 16s rDNA V4 hypervariable region was amplified using primers 515F 5′-GTGCCAGCMGCCGCGGTAA-3′ and 806R 5′-GGACTACHVGGGTWTCTAAT-3′. At the end 2 × 250 bp paired-end reads were generated by sequencing on the Illumina HiSeq 2500 platform. The primers were assessed using PrimerProspector Software Package (see Supplementary Figure 1). H_2_O was used to negative control for sequencing in each sample.

### Sequencing Data Analysis

High-quality data were acquired as the in-house procedure from BGI Co., Ltd., China. Then the clean reads were overlapped to obtain tags using FLASH (v1.2.11) (Magoc and Salzberg, 2011). The clean tags which were dereplicated and filtered singletons to cluster into operational taxonomic units at 97% sequence similarity using USEARCH (v9.1.13) in order to obtain OTUs representative sequences and otu abundance in each sample (Edgar, 2013). Every OTU were assigned to the Greengenes Database (v201305)^1^, at the similarity threshold of 0.5 using RDP Classifier (v2.2) (DeSantis et al., 2006; Wang et al., 2007). Alpha diversity analysis was performed by mothur (v1.31.2) (Schloss et al., 2009). The non-parametric tests were adopted in alpha diversity analysis. Wilcoxon rank sum test was used between two groups, and Kruskal test was used in the comparison of three groups or more than three groups in alpha diversity analysis. Beta diversity analysis were assessed by QIIME (v1.9) (Caporaso et al., 2010). The results of beta diversity were showed by the principle coordinate analysis (PCoA) of weighted and unweighted UniFrac distance. Differences in beta diversity were evaluated by ANOSIM analysis of similarity and Mantel correlation analysis with 999 permutations (Oksanen, 2011).

### Differential Taxonomy Analysis

The comparisons of genera relative abundance were performed by Metastats^2^ and linear discriminant analysis (LDA) effect size (LEfSe) between PN and T groups (White et al., 2009; Segata et al., 2011). The genus with greater than 3 at the base of *P*-value < 0.05 were considered to choose.

### Functional Metagenome Predictions

We predicted the KEGG and COG by Phylogenetic Investigation of Communities by Reconstruction of Unobserved States (PICRUSt, v1.0.0) (Langille et al., 2013), after constructing the close reference of OTU representative sequences from the discovery cohort Qiime software. The accuracy of the predicted metagenome was evaluated by the NSTI value. The predicted KEGG and COG function analysis by STAMP software using two-sided test with Welch’s *t*-test corrected with Benjamini-Hochberg FDR (Parks et al., 2014). The LEfSe analysis was based on the relative abundance of predicted KEGG Pathways.

### Logistic Regression and Receiver Operating Characteristic (ROC) Analyses

Receiver operating characteristic curves were constructed to reflect the discriminatory potential of the microbiota abundance to detect esophageal cancer. ROC curves and *P*-values were analyzed according to Wilson/Brown method recommended by GraphPad Prism v8.0.2. AUC represents the area under the curve, and CI represents the confidence intervals.

### Microbial Dysbiosis Index (MDI)

The MDI was determined as the log of (total abundance of genera increased in Tumor groups) over (total abundance of genera decreased in Tumor groups) (Gevers et al., 2014). Acinetobacter spp., Blastomonas spp., Klebsiella spp., Pseudomonas spp., Lactococcus spp., Thermus spp., Anoxybacillus spp., Geobacillus spp. were included as decreased in Tumor groups, and Campylobacter spp., Fusobacterium spp., Non-Fusobacterium Fusobacteria, Parvimonas spp., Peptostreptococcus spp., Selenomonas spp., Streptococcus spp., Veillonella spp., Prevotella spp., Treponema spp., Capnocytophaga spp. were included as increased in Tumor groups.

## Results

### 16S rRNA Microbiota Profiling in Physiological Normal Esophagus and ESCC

We compared the 16S rRNA gene of the esophageal microbiota between patients with ESCC (T) and patients with physiological normal esophagus (PN) by NGS. After sequencing and quality filtering, more than 1171914 million clean Tags were obtained corresponding to a mean of 310 OTU and clean tags 40410 per sample. The number of clean Tags was not significantly different between PN and T groups (Supplementary Figure 2A). To determine the number of biologically significant OTUs, we classified the OTUs of PN and T groups according to the sequences of the Greengenes Named Isolated database. The results indicated that the frequency of bacteria OTUs was major fraction in OTU classification (Supplementary Figure 2C). Furthermore, Venn diagram displaying the number of common and specific OTUs identified between PN and N groups (Supplementary Figure 2D).

### The Profile of Esophageal Microbiota Demonstrates Difference in Physiological Normal Esophagus and ESCC

**FIGURE 1:**
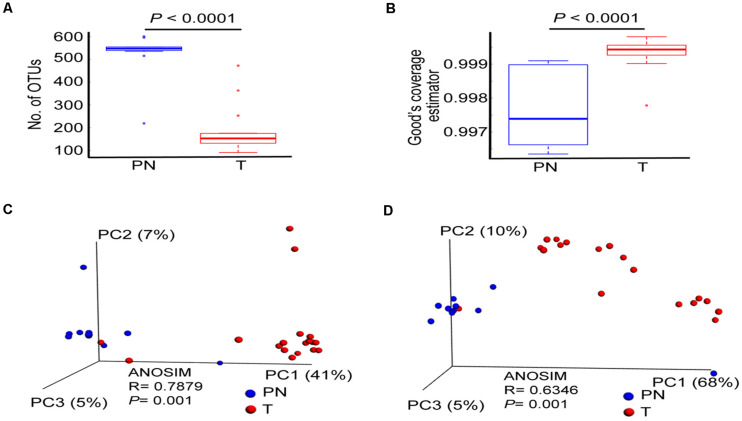
The profile of esophageal microbiota differs in the PN and T groups. **(A)** Number of OTUs in PN and T groups, measuring the total bacterial species diversity in two groups. **(B)** Good’ s coverage Index of diversity, measuring the data coverage rate of total bacterial species in two groups. **(C)** Principal coordinate analysis (PCoA) plots of unweighted UniFrac distances between PN and T groups, including ANOSIM analysis of similarity. **(D)** Principal coordinate analysis (PCoA) plots of weighted UniFrac distances between PN and T groups, including ANOSIM analysis of similarity. PN represents healthy group; T represents tumor groups.

We computed the alpha diversity of microbes of the T and PN using the OUTs, Shannon index, Simpson index and Good’s coverage. On average, patients with ESCC had a significantly lower number of OUTs than PN esophagus (Figure 1A and Supplementary Figure 2F). However, the Shannon index and Simpson index were not significantly different between the two groups (Supplementary Figures 2B,E). To estimate the bacterial diversity, we used Good’s coverage estimator to determine the proportion of total bacterial species represented in samples of each group. Statistical analysis of Good’s coverage showed that T groups had significantly different numbers of species when compared with PN groups (Figure 1B) by measuring beta diversity using both unweighted and weighted UniFrac phylogenetic distance matrices. The microbiota composition of patients with T groups was significantly different from that of PN groups (ANOSIM *R* = 0.7879, *P* = 0.001; and ANOSIM *R* = 0.6346, *P* = 0.001, for unweighted and weighted distances, respectively, Figures 1C,D).

Age is one of the risk factors for ESCC development. Since patients with ESCC were significantly older than patients with PN esophagus in our cohorts (Supplementary Table 1), we next asked whether the microbial profile was different between the two groups. Overall, age factor was not influenced the microbiota profiles of full sample set (Supplementary Figures 3A,B). However, compared with age-matched microbiota in patient with ESCC and PN esophagus using unweighted and weighted UniFrac distance matrices, we found that microbiota composition was significant in the two clinical settings (Supplementary Figures 3C,D). Furthermore, in the age-matched comparisons, the microbial alpha diversity in ESCC patients was dramatic reduced (Supplementary Figure 3E, *p* = 0.001). Intriguingly, in the cancer progress comparisons, we found that patients with ESCC had significantly decreased microbial diversity than patients with PN and PreT (pre-cancer) (Supplementary Figure 4C). The microbiota composition of patients with T groups was significantly different from that of PN and PreT groups (ANOSIM *R* = 0.53029, *P* = 0.001; and ANOSIM *R* = 0.4792, *P* = 0.001, for unweighted and weighted distances, respectively, Supplementary Figures 4F,G). The relative abundance of Fusobacterium spp. gradually increased from PN esophagus to ESCC, while the abundance of Proteobacteria was decreased (Supplementary Figure 4E). However, there are no statistically significant differences in the microbiota profiles of ESCC patients with gender and tumor stage (Supplementary Figures 4A,B,D,H,I). Altogether, these results showed that there are significant decreased in microbial diversity and composition in ESCC.

### The Abundance of Fusobacterium spp. Affects the Microbiota Composition of Physiological Normal Esophagus and ESCC

**FIGURE 2:**
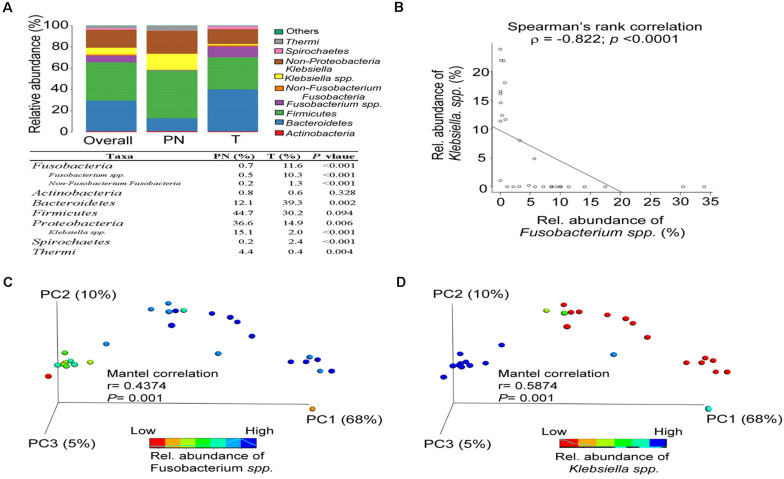
The abundance of *Fusobacterium spp.* affects the microbiota composition of PN and T groups. **(A)** Relative abundance of phyla in all samples and in each group. **(B)** Spearman’s rank correlation between relative abundance of *Fusobacterium spp.* and *Klebsiella spp.* in all samples. **(C)** Principal coordinate analysis (PCoA) plots of the weighted UniFrac distance displayed by increasing the relative abundance of *Fusobacterium spp.* between PN and T groups, including Mantel correlation analysis. **(D)** Principal coordinate analysis (PCoA) plots of weighted UniFrac distances displayed by increasing the relative abundance of *Klebsiella spp.* between PN and T groups, including Mantel correlation analysis.

Overall, the esophageal microbiota was dominated by seven phyla: *Fusobacteria* (7.43%), *Actinobacteria* (0.7%), *Bacteroidetes* (28.93%), *Firmicutes* (35.76%), *Proteobacteria* (23.21%), *Spirochetes* (1.57%), and *Thermi* (1.91%). The ESCC microbiota had an over-representation of *Fusobacteria* (*P* < 0.001), *Bacteroidetes* (*P* = 0.002) and *Spirochaetes* (*P* < 0.001) and lower abundance of *Proteobacteria* (*P* = 0.006) and *Thermi* (*P* = 0.004; Figure 2A). In addition, a significant negative correlation was observed between *Fusobacteria spp.* and *Klebsiella. spp.* (*r* = −0.822, *P* < 0.001; Figure 2B). Accordingly, the microbiota profiles of the two groups could be discriminated by the abundance of *Fusobacterium spp.* (Mantel correlation, *r* = 0.4374, *P* = 0.001; Figure 2C) and *Klebsiella. spp.* (Mantel correlation, *r* = 0.5874, *P* = 0.001; Figure 2D). Altogether, these data indicated that the classification of the esophageal microbial communities differs in ESCC and PN esophagus. Also, our results verify that *Fusobacteria* exists in the ESCC microbiota as a high abundant.

### Characterized Microbial Taxa Associated With Esophageal Carcinoma Patients

**FIGURE 3:**
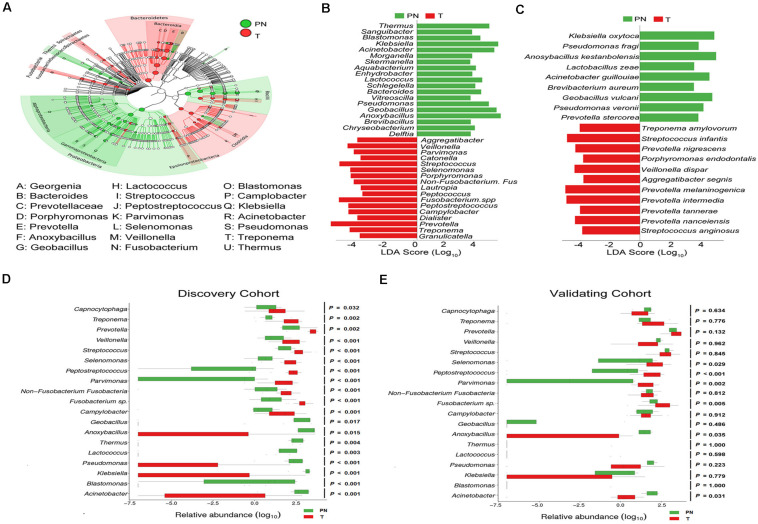
Characterized microbial taxa associated with esophageal carcinoma patients. **(A)** GraPhlAn (Graphical Phylogenetic Analysis) result of the esophageal microbial biomarker between PN and T groups. **(B)** Genus microbial biomarker between PN and T groups by linear discriminant analysis (LDA) effect size (LEfSe). Green represents genus biomarker enriched in PN group and red represents genus biomarker enriched in T group. **(C)** Species microbial biomarker between PN and T groups by linear discriminant analysis (LDA) effect size (LEfSe). **(D)** Relative abundance of the 19 genera differentially enriched between PN and T groups in the Discovery cohort. *P* < 0.05 significance obtained by Metastats software analysis. **(E)** Relative abundance of the 19 genera differentially enriched between PN and T groups in the Validation cohort. *P* < 0.05 significance obtained by Metastats software analysis.

We used LEfSe analysis to identify the relevant taxa responsible for the significances between clinical diagnoses. 31 taxa, including 10 genera, which was differentially abundant between T and PN groups, was identified. Based on Genus taxa in T groups, the enrichment in *Aggregatibacter, Veillonella, Parvimonas, Catonella, Streptococcus, Selenomonas, Porphyromonas, Non-Fusobacterium. Fus, Lautropia, Peptococcus, Fusobacterium*. spp., *Peptostreptococcus, Campylobacter, Dialister, Prevotella, Treponema*, and *Granulicatella* were observed. In addition, *Treponema amylovorum, Streptococcus infantis, Prevotella nigrescens, Porphyromonas endodontalis, Veillonella dispar, Aggregatibacter segnis, Prevotella melaninogenica, Prevotella intermedia, Prevotella tannerae, Prevotella nanceiensis*, and *Streptococcus anginosus* were also significantly more abundant in ESCC by Species taxa (Figures 3A–C).

The age-matched comparisons of the bacteria taxa in patients with ESCC and physiological normal esophagus was performed by LEfSe analysis (Supplementary Figure 3G). To determine the relationships between disease-associated taxa and the abundance of *Fusobacteria*, we subtracted the *Fusobacteria* reads and re-analyzed the library from the dataset by LEfSe analysis. In support of the above, *Streptococcus*, and *Prevotella* in ESCC were enriched (Supplementary Figure 5). To confirm ESCC-enriched and depleted taxa, we used 16s rRNA-seq data from a discovery cohort of ESCC. In this dataset, we found significant decreases in the abundance of *Geobacillus, Anoxybacillus, Thermus, Lactocccus, Pseudomonas, Klebsiella, Blastomonas*, and *Acinetobacter* in ESCC compared to physiological normal esophagus (Figure 3D). To illustrate that our results were not biased by microbiota profiling pipeline, we used a second validation cohort to confirm. In agreement with the results obtained in discovery cohort, the enrichments of *Selenomonas, Peptostreptococcus, Fusobacterium spp.*, and *Acinetobacter* were confirmed in 19 genera as identified by the LEfSe analysis (Figure 3E).

### ESCC Demonstrates Microbial Dysbiosis

**FIGURE 4:**
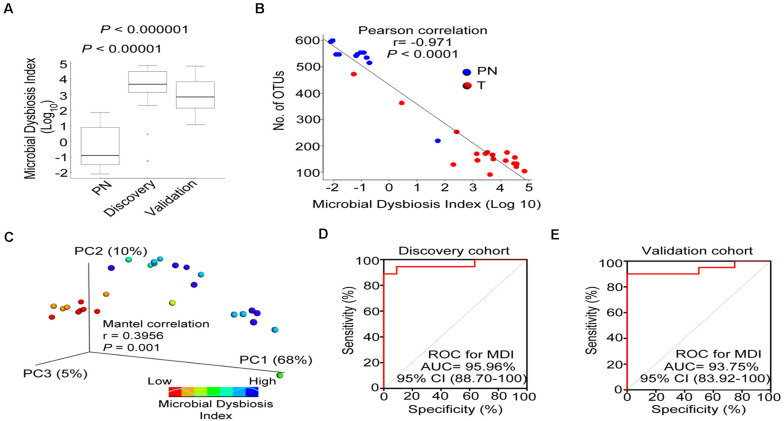
Esophageal carcinoma demonstrates microbial dysbiosis. **(A)** Box plot displaying the MDI in the discovery and validation cohorts. *P*-value was obtained by Wilcox test for comparisons from two groups. **(B)** Negative Pearson’s correlation between MDI and Number of OTUs. **(C)** Principal coordinate analysis (PCoA) plot of weighted UniFrac distance showed by increasing MDI between PN and T groups. Mantel correlation analysis with 999 permutations were used. **(D,E)** ROC curves analysis to assess the discriminatory potential of MDI in the discovery and validation cohorts. MDI, microbial dysbiosis index; ROC, receiver operating characteristic; AUC, area under the curve.

We combined the 19 relevant taxa which characterized in patients with ESCC and PN esophagus and estimated the microbial dysbiosis index (MDI). The esophageal microbiota of patients with ESCC had a higher MDI than that patients with PN esophagus both in discovery cohort and validation cohort (Figure 4A). Moreover, similar results were also observed in age-matched subset of discovery cohort (Supplementary Figure 3F). In Figures 4B,C, we found that MDI had a significant inverse correlation with the alpha diversity (*r* = −0.971, *P* < 0.0001) and a positive correlation with the beta diversity (*r* = 0.3956; *P* < 0.001), indicating that esophageal microbiota has a high degree of dysbiosis, consistent with reduced bacterial diversity. We further assessed if MDI could be use employed to distinguish between ESCC and physiological normal esophagus. By ROC analysis, the MDI had a good performance in identifying ESCC in discovery cohort (AUC = 95.96%, Figure 4D) and validation cohort (AUC = 93.75%, Figure 4E). Additionally, The MDI displayed enhanced sensitivity and specificity to monitor ESCC by using single taxa (Supplementary Figure 6). In validation cohorts (as shown in Figure 3E), we confirmed the differential abundance of Fusobacterium sp., Peptostreptococcus sp., Selenomonas sp. and Acinetobacter sp. We next re-calculated the MDI with these 4 genera. The data showed that microbiota was imbalanced in the discovery cohort, validation cohort and AUCs in the ROC analysis (Supplementary Figure 7).

### Change of Nitrate/Nitrite Reductase Functions in the Microbiota of ESCC

**FIGURE 5:**
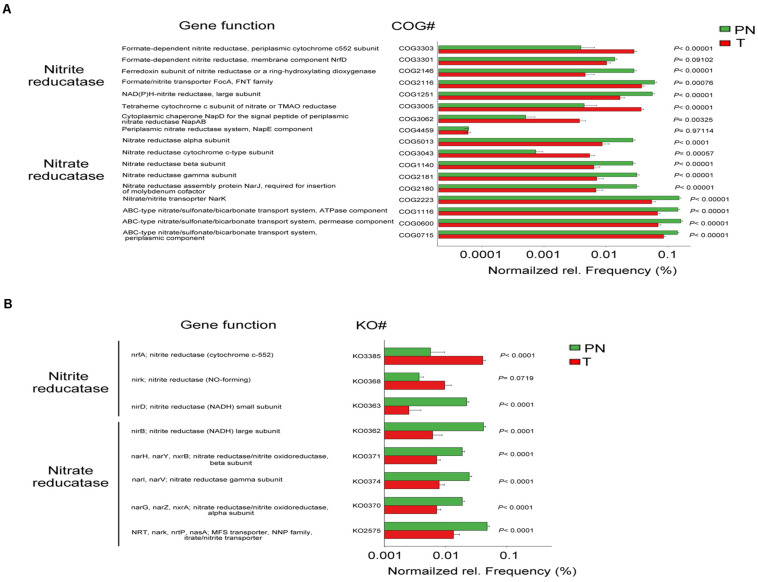
Change of nitrate/nitrite reductase functions in the microbiota of ESCC. **(A)** COG functional analysis of the predicted metagenome of the microbiota between PN and T groups from the discovery cohort. **(B)** KO functional analysis of the predicted metagenome of the microbiota between PN and T groups from the discovery cohort. The normalized relative frequency of nitrate reductase and nitrite reductase in two groups. *P* < 0.05 significance obtained by STAMP software analysis. COG, Clusters of Orthologous Groups; KO, Kyoto Encyclopedia of Genes and Genome (KEGG) orthology; NADH, nicotinamide adenine dinucleotide; NO, nitric oxide; MAO, trimethylamine N-oxide.

The microbial connection in ESCC and PN esophagus could be discriminated according to their function (Supplementary Figure 8A). The predicted KEGG pathways significantly enriched in ESCC included aminoacyl-tRNA biosynthesis, translation proteins, ribosome biogenesis, ribosome, purine metabolism, DNA repair and recombination proteins, DNA replication proteins and Chromosome (Supplementary Figure 8B and Supplementary Table 5). Accumulating evidence demonstrated that the microbiota might produce secondary metabolites, such as reactive nitrate and nitrite, which are carcinogens associated with cancer development. We next compared ESCC and PN esophagus regarding the microbial functional signatures involved in nitrate and nitrite reductase (Supplementary Table 6). The results indicated that the functional composition of ESCC microbiota had decreased nitrate reductase functions and nitrite reductase functions compared to the PN esophagus (Figures 5A,B). Collectively, these data indicated that the change of nitrate and nitrite reductase functions of ESCC microbiota is present in ESCC.

## Discussion

In this study, we profiled the microbial alterations in the tumor and PN tissues of ESCC patients using analyses based on 16S rRNA gene sequencing. We have shown that the microbiota composition in tumor tissues of ESCC patients is significantly different from that of patients with PN tissues. The microbial dysbiosis of ESCC tumor tissues was characterized by decreased microbial diversity, and increased *Bacteroidetes, Fusobacteria*, and *Spirochaetes*. In addition, we also found that the functional features of ESCC microbiota demonstrate reduced nitrate reductase and nitrite reductase functions. Together, our results explored the microbiota spectrum of ESCC patients and revealed the microbial signature associated with ESCC for diagnosis of patients.

The microbiota is less characterized in ESCC than in esophageal adenocarcinoma (Di Pilato et al., 2016; Nardone et al., 2017). Only a few studies have the composition of microbiota on ESCC. Here, we focused on the pathological microbiota characterization in ESCC. The composition of the microbiota of the ESCC has been identified in our study. We found that *Treponema amylovorum, Streptococcus infantis, Prevotella nigrescens, Porphyromonas endodontalis, Veillonella dispar, Aggregatibacter segnis, Prevotella melaninogenica, Prevotella intermedia, Prevotella tannerae, Prevotella nanceiensis*, and *Streptococcus anginosus* are enriched in ESCC tissues. Also, our data indicated that in comparison with physiological normal tissues, a significantly lower microbial richness and alpha diversity in ESCC tumor tissues were observed in our testing and validating cohorts. In addition, the esophageal microbiota of patients with ESCC had a higher MDI. Noticeably, the microbiota composition of patients with PreT (pre-cancer) groups was significantly different from that of tumor groups, suggesting that these unusual microbiota could be diagnostic factor for ESCC development. However, Shao’s study showed no significant difference in alpha diversity for ESCC tumor and non-tumor tissues and *Fusobacterium* were enriched in the esophagus of patients with ESCC (Shao et al., 2019). The discrepancy may be due in part to different variables affecting gut microbiome composition such as geographic area. In our studies, *Fusobacterium nucleatum*, also called by a recently coined name *oncobacterium* because of its association with cancer (Brennan and Garrett, 2019), is again abundant in ESCC tissues of our cohorts. Similar results were also observed in Japanese patients with esophageal cancer (Yamamura et al., 2019). Our detection of these 10 bacterial strains are mainly involved in oral health, suggesting that oral hygiene may have roles in tumor development of ESCC. Indeed, lifestyle factors, including diet, obesity, alcohol and tobacco use, and oral hygiene, are involved in high rates of ESCC. In line with this hypothesis, it has been shown that numbers of lost teeth increase the risk of ESCC development (Chen et al., 2017). Our detection of these 10 bacterial strains are mainly involved in oral health, suggesting that oral hygiene may have roles in tumor development of ESCC. Indeed, lifestyle factors, including diet, obesity, alcohol and tobacco use, and oral hygiene, are involved in high rates of ESCC. In line with this hypothesis, it has been shown that numbers of lost teeth increase the risk of ESCC development (Chen et al., 2017) and that high burden of *F. nucleatum*, which inhabits the oral cavity and causes periodontal disease, in ESCC correlates with poor RFS (Yamamura et al., 2019). FadA, a cell surface protein, is expressed in the *F. nucleatum in vitro* and *in vivo* data demonstrated that FadA could activate Wnt/b-catenin signaling to promote cell growth via binding to E-cadherin in human cancer (Rubinstein et al., 2013). In Apc^*Min/+*^ mouse model, *F. nucleatum* exerted immunosuppressive activity to Inhibit immune response-mediated by T cells to facilitate tumor progression (Mima et al., 2015). Recent study demonstrated that colonization by *F. nucleatum* provoked the secretion of immune cytokines, resulting in colon tumorigenesis (Kostic et al., 2013). Another study showed that CCL20 was upregulated in *F. nucleatum*-positive esophageal cancer patients (Yamamura et al., 2016). CCL20 plays crucial roles in promoting cell proliferation and migration in human cancers (Wang et al., 2016). Collectively, these results suggest that *F. nucleatum* might contribute to the aggressive tumor behavior via the activation of cytokine.

The Diet and medications can affect the composition of microbiota in esophagus. Nobel et al. (2018) recently reported that a high-fiber diet correlated with overabundance of Firmicutes, however, a low-fiber diet was associated the increased abundance of Prevotella and Neisseria (Gyawali et al., 2020). Accumulating evidence showed that esophageal microbiome was altered in person with proton pump inhibitor (PPI) used. Amir et al. observed that Clostridia was significant increase in the esophagus of men with PPI therapy after 8 weeks (Snider et al., 2019). Previous studies also showed that host factors, such as genetic background and age have influence the microbial composition of esophagus. Deshpande et al. (2018) found dramatically changes in the microbial communities of *Streptococcus* and *Prevotella* that correlated with human’s age. In addition, they also demonstrated that host SNP genes, such as NERP and Notch2 which involved in TGF-b and Notch signaling pathway may influence the esophageal microbiome. Taken together, diet, medication, age and genetic background may play important signatures to monitor the composition of microbiota in esophagus.

Here our data further characterize top 10 bacteria strains that may influence or directly participate in carcinogenesis and progression of ESCC. *Treponema amylovorum* is a subgingival plaque bacteria species involved in chronic periodontitis (Karasneh et al., 2017). *Streptococcus infantis* is *a* part of the salivary microbiome (Kilian and Tettelin, 2019) and a prevalent bacteria in breast cancer (Nejman et al., 2020). *Aggregatibacter segnis* is an oral bacterial species in oral cancer (Zhang et al., 2019). *Porphyromonas endodontalis* is *a* part of the salivary microbiome, and its lipopolysaccharide (LPS), which can trigger NFκB signaling (Yu et al., 2015), is enriched in infected root canals and apical periodontitis (Guo et al., 2014). *Veillonella dispar*, a bacterium in oral mucosa, is involved in autoimmune hepatitis (Wei et al., 2020; Yuan et al., 2020). *Streptococcus anginosus* is enriched in gastric cancer (Coker et al., 2018) and dental implant-related osteomyelitis (Chatelain et al., 2018). *Prevotella intermedia* is a biomarker for periodontal disease (Zhang et al., 2017). *Prevotella melaninogenica* is a causative agent of periodontitis (Vuotto et al., 2014). *Prevotella nigrescens*, which elicits TLR2 signaling and p65-Mediated Inflammation (Bertelsen et al., 2020), is an oral bacterial species causing for respiratory tract infections. Further, *Prevotella intermedia, Prevotella melaninogenica*, and *Prevotella nanceiensis* are three periodontopathic bacteria species causing oral malodor and oral health issues (Yanagisawa et al., 2006). They have cysteine and serine proteinases activity that may regulate tumorigenesis (Yanagisawa et al., 2006). *Porphyromonas gingivalis*, a Gram-negative bacterial species involved in periodontal diseases, is a biomarker of ESCC (Malinowski et al., 2019). Lastly, *Prevotella tannerae*, a periodontopathogenic bacteria, is associated with an increased oral squamous cell carcinoma risk (Hsiao et al., 2018). The resident microbial ecosystem in the esophagus is affected by both oral and gastric bacteria, but our data show that microbiota of ESCC seems to be dominated by oral bacteria. As mentioned above, top enrichments in microbial composition of ESCC are mainly from oral bacteria strains, we conclude that oral microbial dysbiosis leads to the occurrence and development of ESCC. These data suggest that poor oral health is linked to increased risk of developing ESCC. We speculate that these specific bacterial strains in oral samples might be utilized as screening tools to assess the risk for ESCC to identify high risk individuals for more invasive screening procedures (e.g., endoscopy). It is possible that the altered abundances of these bacterial strains have a causative role in ESCC development. Their detailed mechanisms in promoting ESCC require further investigation.

We have depicted the diversity of the gut microbiota in ESCC, but the role of most bacterial species in ESCC remains largely unknown. The complexity of the ESCC microbiota, with a plethora of uncharacterized host-microbe, microbe-microbe, and environmental interactions, contributes to the challenge of advancing our knowledge of the ESCC microbiota-cancer interaction. To address the microbiota-cancer interaction, we have investigated KEGG pathways that were enriched in ESCC-associated bacteria. One of the top-ranked KEGG pathway in ESCC microbiota is nitrate reductase function. The nitrate reductase might transform nitrate to nitrite, a precursor of nitrosamines, which are carcinogens associated with ESCC. Nitrate is critical inorganic nitrogen sources for microbes, and many bacteria express assimilatory nitrate reductase to catalyze the rate-limiting reduction of nitrate to nitrite. For examples, it is interesting to note that fast-growing environmental mycobacteria carry *nasN*, while slow-growing pathogenic mycobacteria are lacking (Tan et al., 2020). Also, it has been shown that nitrite-oxidizing phylum Nitrospirae is reduced in gastric cancer (Wang et al., 2020), causing decreased nitrate/nitrite reductase functions. These data suggest that nitrate reduction plays role in pathogenic/neoplastic progression. And our observation that reduction of nitrate reductase functions in the microbiota of ESCC may impose pathogenic effects during ESCC progression and development. Given that ESCC-associated taxa can impact the nitrate regulation, it is possible that targeting the microbiota involved in nitrate regulation may be effective and beneficial to ESCC patients.

A recent comprehensive investigation of microbiomes across seven cancer types (not yet including ESCC) indicates that intracellular bacteria are widespread in tumors (Nejman et al., 2020). Particularly, 19 prevalent bacteria are characterized (Nejman et al., 2020), including genera of streptococcus and fusobacterium, which are found in our ESCC microbiota. It is not clear whether the enriched bacterial species or genera identified in our study can reside in ESCC to facilitate the microenvironment to boost cancer growth. These species may impose immune inflammatory and metabolic burden, and further studies are warranted. ESCC is one of the most aggressive cancers and is therapeutic resistant including radioresistance (Li et al., 2020). The dysbiosis of esophageal microbiota could be the culprit of these impacts (Yamamura et al., 2019), suggesting the possibility that a strategic intervention against this bacterium may significantly improve therapeutic response in patients with ESCC. Indeed, microbiota composition can influence the treatment efficacy of immune checkpoint inhibitor in melanoma (Gopalakrishnan et al., 2018; Inamura, 2020). As immune checkpoint inhibitors (e.g., nivolumab, or pembrolizumab) are being used to treat ESCC (Smyth et al., 2017), unraveling ESCC microbiota-drug interactions and efficacy warrants further investigation.

In summary, our studies suggest that ESCC-specific microbial taxa may serve as sensitive and specific clinical diagnostic markers. It is possible that targeting these bacterial strains may be effective and beneficial to ESCC patients. Correct microbial assessment will aid in the detection and treatment of ESCC in the future.

## Data Availability Statement

The raw sequence data reported in this paper have been deposited in the Genome Sequence Archive (Genomics, Proteomics & Bioinformatics 2017) in National Genomics Data Center (Nucleic Acids Res 2021), China National Center for Bioinformation/Beijing Institute of Genomics, Chinese Academy of Sciences, under accession number HRA000651 that are publicly accessible at: http://bigd.big.ac.cn/gsa-human.

## Ethics Statement

This study was conducted according to a protocol approved by the respective Institutional Ethics Committees of The First Affiliated Hospital of Sun Yat-sen University (ChiCTR1800018897) and according to the Declaration of Helsinki. The patients/participants provided their written informed consent to participate in this study.

## Author Contributions

CC and M-HL: study design. WY, XX, ZZ, ZL, and BZ: sample collection and processing. WY, C-HC, MJ, ZZ, ZL, and BZ: clinical data collection and interpretation. WY, C-HC, MJ, ZZ, and LG: bioinformatics analysis and statistics. WY, C-HC, LG, SY, CC, and M-HL: manuscript preparation. WY, C-HC, MJ, XX, LG, ZZ, ZL, BZ, SY, M-HL, and CC: approval of final draft submission. All authors contributed to the article and approved the submitted version.

## Conflict of Interest

LG was the employee of BGI Genomics, BGI-Shenzhen, China. The remaining authors declare that the research was conducted in the absence of any commercial or financial relationships that could be construed as a potential conflict of interest.

## Footnotes

1 http://greengenes.secondgenome.com/downloads

2 http://metastats.cbcb.umd.edu/

## Supplementary Material

Supplementary Figure 1 Performance of the primers (515F and 806R) for 16S rRNA V4 Illumina HiSeq 2500 sequencing evaluated by PrimerProspector software package. **(A,B)** Number of sequences matched by the forward and reverse primer. **(C,D)** Wrong reads matched *Bacteria* and *Archaea.* **(E)** Taxonomic coverage of simulated reads by taxonomic level.

Supplementary Figure 2 16S rRNA V4 reads derived from 11 healthy people and 18 tumor esophageal carcinoma patients. **(A)** Number of clean tags. **(B)** Simpson Index of diversity, measuring the data evenness of total bacterial species in two groups. **(C)** Pie chart displaying OTUs classified according to the result of comparing the Greengenes Database; OTUs using usearch (usearch9.1.13_i86linux32) to cluster on the level of 97% similarity were acquired. **(D)** Venn diagram displaying the number of common and specific OTUs, respectively, in two groups. **(E)** Shannon’s Index of diversity, measuring the data evenness of total bacterial species in two groups. **(F)** Rarefaction curves of the number of OTUs versus from the samples of discovery cohort.

Supplementary Figure 3 **(A)** Principal coordinate analysis (PCoA) plots of unweighted UniFrac distances in the all samples of discovery cohort as increasing age. Mantel correlation analysis with 999 permutations were used. **(B)** Principal coordinate analysis (PCoA) plots of weighted UniFrac distances in the all samples of discovery cohort as increasing age. Mantel correlation analysis with 999 permutations were used. **(C)** Principal coordinate analysis (PCoA) plots of unweighted UniFrac distances in age-matched healthy group (*n* = 9) and tumor group (*n* = 9), including ANOSIM analysis of similarity. **(D)** Principal coordinate analysis (PCoA) plots of weighted UniFrac distances in age-matched healthy group (*n* = 9) and tumor group (*n* = 9), including ANOSIM analysis of similarity. **(E)** Box plot displaying the number of OTUs in PN and T groups which are age matched. **(F)** LEfSe analysis displaying specific microbial biomarker in PN and T groups which are age matched. **(G)** Box plot displaying the MDI in PN and T groups which are age matched.

Supplementary Figure 4 **(A)** Principal coordinate analysis (PCoA) plots of unweighted UniFrac distances between Female and Male groups, including ANOSIM analysis of similarity. **(B)** Principal coordinate analysis (PCoA) plots of weighted UniFrac distances between Female and Male groups, including ANOSIM analysis of similarity. **(C)** Box plot displaying the number of OTUs according to tumor existence and location. **(D)** Box plot displaying the number of OTUs according to tumor development stages in tumor groups. **(E)** Relative abundance of phyla in all samples. which are grouped according to tumor existence and location. **(F)** Principal coordinate analysis (PCoA) plots of unweighted UniFrac distances according to tumor existence and location, including ANOSIM analysis of similarity. **(G)** Principal coordinate analysis (PCoA) plots of weighted UniFrac distances according to tumor existence and location, including ANOSIM analysis of similarity. **(H)** Principal coordinate analysis (PCoA) plots of unweighted UniFrac distances according to tumor development stages in T groups. **(I)** Principal coordinate analysis (PCoA) plots of weighted UniFrac distances according to tumor development stages in T groups.

Supplementary Figure 5 LEfSe reanalysis when *Fusobacterium spp.* was excluded from the differential results of discovery cohort.

Supplementary Figure 6 ROC curves analysis to assess the discriminatory potential of the microbiota abundance in the validation cohort. **(A)** *Fusobacterium* spp. **(B)** *Peptostreptococcus* spp. **(C)** *Prevotella* spp. **(D)** *Streptococcus* spp. **(E)** *Treponema* spp. **(F)** *Veillonella* spp. **(G)** *Acinetobacter* spp. **(H)** *Klebsiella* spp.

Supplementary Figure 7 Microbial dysbiosis index calculated remaining *Fusobacterium sp., Peptostreptococcus sp., Selenomonas sp.*, and *Acinetobacter sp.* **(A)** Box plot displaying the MDI in the discovery cohort. *P*-value was obtained by Wilcox test. **(B)** Box plot displaying the MDI in the validation cohort. *P*-value was obtained by Wilcox test. **(C)** ROC curves analysis to assess the discriminatory potential of MDI in the discovery cohort. **(D)** ROC curves analysis to assess the discriminatory potential of MDI in the validation cohort.

Supplementary Figure 8 **(A)** Principal coordinate analysis (PCA) plots of environmental function predictions at the level of COG and KO by PICRUSt software between PN and T groups. **(B)** The figure displaying the correlation of predicted function of L2 level compared KEGG database and the biomarker genus from MDI.

## Abbreviations

ESCC: esophageal squamous cell carcinoma
KEGG: Kyoto Encyclopedia of Genes and Genomes
MDI: Microbial dysbiosis index
NGS: next-generation sequencing
PN: physiological normal
ROC: receiver operating characteristic.
