# Supplementary material for: Tumor-Associated Microbiota in Esophageal Squamous Cell Carcinoma
Source: Front Cell Dev Biol. 2021 Feb 18;9:641270. doi: 10.3389/fcell.2021.641270 (PMC7930383; doi:10.3389/fcell.2021.641270)
Supplement: Supplementary file 1 [file Data_Sheet_1.PDF]

Supplementary Table S1. Characteristics of the patients in the discovery and validation cohorts.

**Patient characteristics**	**Discovery cohort**			**Validation cohort**		
	**Physiological normal（*n*=11）**	**Esophageal carcinoma （*n*=18）**	***P* Value**	**Physiological normal（*n*=4）**	**Esophageal carcinoma （*n*=20）**	***P* Value**
Age (mean±SD)	49.91±13.42	62.83±10.18	**0.007**	41.25±16.99	61.25±7.37	**0.001**
Gender						
Male	6	13	0.33	3	14	1.00
Female	5	5		1	6	
BMI	23.0±1.8	21.2±2.5	**0.048**	24.83±4.40	21.92±3.25	0.137
Smoking status						
Non-smokers	5	9	0.812	3	15	1.00
Smokers	6	9		1	5	
Alcohol consumption						
Non-drinkers	5	14	0.114	2	16	0.251
Drinkers	6	4		2	4	
Family history of ESCC						
Non-family history	11	17	1.00	4	20	1.00
Family history	0	1		0	0	
T stage						
T1	NA	0		NA	2	
T2	NA	3		NA	3	
T3	NA	14		NA	13	
T4	NA	1		NA	2	
Lymph node metastasis						
Yes	NA	6		NA	11	
No	NA	12		NA	9	
Vascular invasion						
Yes	NA	1		NA	5	
No	NA	17		NA	15	
Stage						
I-II	NA	10		NA	9	
III-IV	NA	8		NA	11	

NA, not applicable. [*]P-value obtained by Fisher's exact test for categorical variables and by t test for continuous variables. Statistical tests were performed two-sided.

Supplementary Table S2. Characteristics of the patients and retrieval data in the discovery and validation cohorts.

Sample ID	Clinical setting	Cohort	Gender	Age	Lymph node metastasis	Vascular invasion	TNM stage
16PN	Physiological normal	Discovery	Female	48	NA	NA	NA
21PN	Physiological normal	Discovery	Male	52	NA	NA	NA
22PN	Physiological normal	Discovery	Male	47	NA	NA	NA
33PN	Physiological normal	Discovery	Male	75	NA	NA	NA
37PN	Physiological normal	Discovery	Female	40	NA	NA	NA
45PN	Physiological normal	Discovery	Male	48	NA	NA	NA
46PN	Physiological normal	Discovery	Female	54	NA	NA	NA
58PN	Physiological normal	Discovery	Female	49	NA	NA	NA
60PN	Physiological normal	Discovery	Male	64	NA	NA	NA
68PN	Physiological normal	Discovery	Male	21	NA	NA	NA
69PN	Physiological normal	Discovery	Female	51	NA	NA	NA
1T	Esophageal carcinoma	Discovery	Female	56	No	No	T3N0M0, IIB
2T	Esophageal carcinoma	Discovery	Male	74	No	No	T2N0M0, IIA
3T	Esophageal carcinoma	Discovery	Male	70	No	No	T3N0M0, IIA
4T	Esophageal carcinoma	Discovery	Female	68	No	No	T3N0M0, IIA
6T	Esophageal carcinoma	Discovery	Male	67	No	No	T3N1M0, IIIB
7T	Esophageal carcinoma	Discovery	Female	70	Yes	No	T3N1M0, IIIB
8T	Esophageal carcinoma	Discovery	Male	43	No	No	T2N0M0, IIA
9T	Esophageal carcinoma	Discovery	Male	65	Yes	No	T3N1M0, IIIB
10T	Esophageal carcinoma	Discovery	Male	46	Yes	No	T3N2M0, IIIB
11T	Esophageal carcinoma	Discovery	Female	62	Yes	No	T3N1M0, IIIB
14T	Esophageal carcinoma	Discovery	Male	69	Yes	No	T3N1M0, IIIB
16T	Esophageal carcinoma	Discovery	Female	78	No	No	T4aN0M0, IIIB
18T	Esophageal carcinoma	Discovery	Male	53	Yes	No	T3N2M0, IIIB
20T	Esophageal carcinoma	Discovery	Male	67	No	No	T3N0M0, IIA
21T	Esophageal carcinoma	Discovery	Male	72	No	Yes	T3N0M0, IIB
22T	Esophageal carcinoma	Discovery	Male	68	No	No	T3N0M0, IIB
23T	Esophageal carcinoma	Discovery	Male	49	No	No	T2N0M0, IIA
25T	Esophageal carcinoma	Discovery	Male	54	No	No	T3N0M0, IIA
73PN	Physiological normal	Validation	Female	57	NA	NA	NA
77PN	Physiological normal	Validation	Male	54	NA	NA	NA
79PN	Physiological normal	Validation	Male	32	NA	NA	NA
80PN	Physiological normal	Validation	Male	22	NA	NA	NA
27T	Esophageal carcinoma	Validation	Male	52	Yes	No	T4bN2M0, IVA
28T	Esophageal carcinoma	Validation	Male	66	Yes	No	T3N1M0, IIIB
30T	Esophageal carcinoma	Validation	Female	59	Yes	No	T3N1M0, IIIB
31T	Esophageal carcinoma	Validation	Male	58	No	Yes	T3N0M0, IIA
32T	Esophageal carcinoma	Validation	Female	67	No	No	T2N0M0, IIA

33T	Esophageal carcinoma	Validation	Male	57	Yes	Yes	T3N1M0, IIIB
34T	Esophageal carcinoma	Validation	Male	63	No	No	T2N0M0, IIA
35T	Esophageal carcinoma	Validation	Female	61	Yes	No	T4bN1M0, IVA
36T	Esophageal carcinoma	Validation	Female	66	No	No	T1aN0M0, IB
37T	Esophageal carcinoma	Validation	Male	61	No	No	T1bN0M0, IB
38T	Esophageal carcinoma	Validation	Male	59	No	No	T3N0M0, IIA
39T	Esophageal carcinoma	Validation	Female	78	No	Yes	T3N0M0, IIA
41T	Esophageal carcinoma	Validation	Female	70	No	No	T3N0M0, IIB
43T	Esophageal carcinoma	Validation	Male	71	No	No	T3N0M0, IIA
44T	Esophageal carcinoma	Validation	Male	51	Yes	No	T3N1M0, IIIB
51T	Esophageal carcinoma	Validation	Male	55	Yes	No	T2N1M0, IIIA
52T	Esophageal carcinoma	Validation	Male	55	Yes	No	T3N1M0, IIIB
53T	Esophageal carcinoma	Validation	Male	48	Yes	Yes	T3N1M0, IIIB
55T	Esophageal carcinoma	Validation	Male	63	Yes	Yes	T3N1M0, IIIB
56T	Esophageal carcinoma	Validation	Male	65	Yes	No	T3N2M0, IIIB

NA, not applicable.

Supplementary Table S3. Assessment effects in the discovery cohort all over the whole experiment process*.

Assessment effects	ROC curves[#] AUC (95% CI)	*P* - values
Alpha diversity (Shannon index)	0.98 (0.95 — 1.00)	< 0.0001
Beta diversity[&]		
Unweighted UniFrac	0.98 (0.95 — 1.00)	< 0.0001
Weighted UniFrac	0.89 (0.72 — 1.00)	< 0.0001
Relative abundance of major phyla		
Proteobacteria	0.79 (0.60 — 0.97)	0.010
Firmicutes	0.61 (0.37 — 0.84)	0.345
Bacteroidetes	0.87 (0.69 — 1.00)	0.001
Fusobacteria	0.98 (0.94 — 1.00)	< 0.0001
Actinobacteria	0.69 (0.49 — 0.88)	0.096
Spirochaetes	0.82 (0.66 — 0.98)	0.005
Thermi	0.90 (0.77 — 1.00)	< 0.0001

ROC, Receiver Operating Characteristic curve; AUC, Area Under the Curve; CI, Confidence intervals.

*The 16S rDNA tags were clustered using USEARCH (v9.1) after filtering (low qlaility reads, N reads) and overlapping.

[#]ROC curves and P-values were analysed according to Wilson/Brown method recommended by GraphPad Prism v8.0.2. Good reproducibility is showed by AUC results.

[&]Beta diversity used the first principal coordinate from weighted UniFrac and unweighted UniFrac distance matrix

Supplementary Table S4. Assessment effects in the discovery cohort all over the whole experiment process.

	PN group (ρ* and *P* - value)	T group (ρ* and *P* - value)
Non-*Fusobacterium Fusobacteria*	0.81 (*P* = 0.0040)	0.16 (*P* = 0.5193)
Actinobacteria	0.13 (*P* = 0.7138)	-0.41 (*P* = 0.0926)
Bacteroidetes	0.15 (*P* = 0.6535)	0.15 (*P* = 0.5578)
Firmicutes	-0.13 (*P* = 0.7138)	-0.37 (*P* = 0.1308)
Proteobacteria	0.15 (*P* = 0.6734)	-0.24 (*P* = 0.3308)
Spirochaetes	0.51 (*P* = 0.1140)	0.16 (*P* = 0.5193)
Thermi	0.03 (*P* = 0.9460)	-0.49 (*P* = 0.0372)

ρ*, Spearman's rank coefficient.

Supplementary Table S5. KEGG Pathways analysis between PN and T groups from the discovery cohort.

KEGG Pathways	PN: mean rel. freq. (%)	T: mean rel. freq. (%)	*P*-values* (corrected)	LDA score	*P*-value (LEfSe)	Enriched Group
1,1,1-Trichloro-2,2-bis(4-chlorophenyl) ethane (DDT) degradation	0.001439242	0.002468738	0.350690632		-	—
ABC transporters	4.588133933	3.039902698	0.00193889	3.88372162	0.000384083	PN
Adherens junction	0	0	1.031446541		-	—
Adipocytokine signaling pathway	0.052186307	0.0849528	0.005313605	2.22340863	0.000635609	T
African trypanosomiasis	0.013801645	0.002736428	0.005461205		3.54E-05	—
Alanine, aspartate and glutamate metabolism	0.772299397	0.95781671	0.007992008	2.975244824	0.000384083	T
Aldosterone-regulated sodium reabsorption	0.001362537	0.000256787	0.011411474		0.000241935	—
alpha-Linolenic acid metabolism	0.044727824	0.011321708	0.00196211	2.223618477	9.22E-05	PN
Alzheimer's disease	0.063993042	0.080329991	0.005553768		0.000538388	—
Amino acid metabolism	0.326287011	0.18683039	0.019274843	2.837000768	7.64E-05	PN
Amino acid related enzymes	1.093400499	1.601321413	0.002730603	3.397376676	4.31E-05	T
Amino sugar and nucleotide sugar metabolism	1.13799073	1.385355061	0.003150695	3.102196166	0.00045517	T
Aminoacyl-tRNA biosynthesis	0.74433446	1.376411685	0.025205564	3.49388503	2.91E-05	T
Aminobenzoate degradation	0.284793947	0.163295082	0.004095904	2.775893112	0.000635609	PN
Amoebiasis	0.00623721	0.007287393	0.664269583		-	—
Amyotrophic lateral sclerosis (ALS)	0.044716146	0.018206435	0.002374437	2.130220525	0.000159675	PN
Antigen processing and presentation	0.019385896	0.032148803	0.00857781		0.002600237	—
Apoptosis	0.003419702	0.006219842	0.163133004		-	—
Arachidonic acid metabolism	0.07050147	0.074886356	0.620236906		-	—
Arginine and proline metabolism	1.188461834	0.872081307	0.011299046	3.190837859	6.33E-05	PN
Arrhythmogenic right ventricular cardiomyopathy (ARVC)	0	0	1.044585987		-	—
Ascorbate and aldarate metabolism	0.372035233	0.115510049	0.023405166	3.105349865	6.33E-05	PN
Atrazine degradation	0.057110517	0.013053558	0.003212474	2.344314223	2.91E-05	PN
Bacterial chemotaxis	0.283577924	0.213978142	0.003810143	2.489273226	0.013433465	PN
Bacterial invasion of epithelial cells	4.25E-05	0.000497859	0.009362067		3.54E-05	—
Bacterial motility proteins	0.803852618	0.497998805	0.002409355	3.16778243	0.00032348	PN

Bacterial secretion system	0.999564094	0.722571671	0.005041113	3.140328439	0.000384083	PN
Bacterial toxins	0.043767831	0.113694734	0.003006168	2.544138189	6.33E-05	T
Basal transcription factors	0.000342767	0.002293389	0.006971752		0.000635609	—
Base excision repair	0.320972441	0.479731288	0.002357355	2.887056426	4.31E-05	T
Benzoate degradation	0.533329127	0.22620144	0.009637421	3.178078327	0.000110902	PN
beta-Alanine metabolism	0.358336335	0.205054689	0.003309821	2.876206787	0.000110902	PN
beta-Lactam resistance	0.016548002	0.007077691	0.002978839		3.55E-05	—
Betalain biosynthesis	0.000303646	6.50E-05	0.00848892		0.002181742	—
Bile secretion	0.000124823	0.001038329	0.003120689		0.004631004	—
Biosynthesis and biodegradation of secondary metabolites	0.191750949	0.050236484	0.013653014	2.847213903	2.39E-05	PN
Biosynthesis of 12-, 14- and 16-membered macrolides	3.03E-06	3.04E-06	1.159097131		-	—
Biosynthesis of ansamycins	0.1426604	0.087961728	0.002114015	2.428677541	1.30E-05	PN
Biosynthesis of siderophore group nonribosomal peptides	0.170350773	0.047037925	0.003276723	2.78462618	1.96E-05	PN
Biosynthesis of type II polyketide backbone	5.32E-06	3.11E-06	0.663024476		-	—
Biosynthesis of type II polyketide products	0.000608165	5.61E-05	0.002244331		5.92E-05	—
Biosynthesis of unsaturated fatty acids	0.250208039	0.111574289	0.046810333	2.839110898	2.39E-05	PN
Biosynthesis of vancomycin group antibiotics	0.025538892	0.070297386	0.002060832	2.349955481	0.00032348	T
Biotin metabolism	0.125724677	0.16469263	0.032767233	2.272215858	0.000635609	T
Bisphenol degradation	0.032249362	0.053021269	0.005342484	2.043742448	0.002600237	T
Bladder cancer	0.012893575	0.002144952	0.003523358		4.30E-05	—
Butanoate metabolism	0.888574374	0.598017554	0.003996004	3.158016244	0.000228141	PN
Butirosin and neomycin biosynthesis	0.027292774	0.051210163	0.007281607	2.091260408	0.000384083	T
C5-Branched dibasic acid metabolism	0.285095916	0.251091885	0.008533134	2.230785777	0.005324894	PN
Caffeine metabolism	0.00014373	5.20E-05	0.074603967		0.011259083	—
Calcium signaling pathway	6.80E-07	5.17E-07	1.000003035		-	—
CAM ligands	0	0	1.142857143		-	—
Caprolactam degradation	0.158624826	0.033605823	0.010570075	2.793982244	7.64E-05	PN
Carbohydrate digestion and absorption	0.030423382	0.030121639	1.037236145		-	—
Carbohydrate metabolism	0.195212484	0.110960639	0.004368964	2.606932146	2.91E-05	PN
Carbon fixation in photosynthetic organisms	0.591352032	0.679409988	0.003485876	2.648468616	0.004631004	T
Carbon fixation pathways in prokaryotes	0.957892084	1.101747363	0.003854969	2.85478621	0.00045517	T
Cardiac muscle contraction	0.007812787	0.016526713	0.091342286		-	—
Carotenoid biosynthesis	0.006285027	0.004371796	0.405002997		-	—

Cell cycle	0	0	1.146853147		-	—
Cell cycle - Caulobacter	0.304803504	0.601505842	0.327672328	3.165347763	5.23E-05	T
Cell cycle - yeast	0	0	1.089700997		-	—
Cell division	0.073194774	0.07532872	0.832164062		-	—
Cell motility and secretion	0.195313279	0.243360018	0.008856009	2.37209889	0.000159675	T
Cellular antigens	0.061524748	0.072542578	0.143267987		-	—
Chagas disease (American trypanosomiasis)	0.012702844	0.002620366	0.002047952		3.55E-05	—
Chaperones and folding catalysts	0.924349038	1.202937459	0.001998002	3.13109266	0.000538388	T
Chloroalkane and chloroalkene degradation	0.208205507	0.119730475	0.163836164	2.64359821	0.00032348	PN
Chlorocyclohexane and chlorobenzene degradation	0.040457176	0.011053754	0.003181285	2.166951995	0.00027192	PN
Cholinergic synapse	0	0	1.047923323		-	—
Chromosome	1.260315348	1.723111604	0.001916212	3.357124315	6.33E-05	T
Chronic myeloid leukemia	1.51E-06	3.05E-07	0.401662853		-	—
Circadian rhythm - plant	0.004028113	0.000516903	0.007801722		0.001116341	—
Citrate cycle (TCA cycle)	0.779970624	0.738768315	0.029506039		-	—
Clavulanic acid biosynthesis	3.46E-06	1.18E-06	0.360035027		-	—
Colorectal cancer	0.002104458	0.000704204	0.041731853		0.015219242	—
Complement and coagulation cascades	0	0	1.131034483		-	—
Cyanoamino acid metabolism	0.281093734	0.21190916	0.002445316	2.517400456	1.30E-05	PN
Cysteine and methionine metabolism	0.938053162	0.893370984	0.017668606	2.30299249	0.019427426	PN
Cytochrome P450	0.000425213	8.75E-06	0.002427202		0.000587041	—
Cytokine receptors	0	0	1.108108108		-	—
Cytokine-cytokine receptor interaction	0	0	1.127147766		-	—
Cytoskeleton proteins	0.158019537	0.263958806	0.003640804	2.73410376	0.00045517	T
Cytosolic DNA-sensing pathway	0	0	1.068403909		-	—
D-Alanine metabolism	0.081057974	0.148640712	0.008191808	2.524010016	1.30E-05	T
D-Arginine and D-ornithine metabolism	0.004117521	0.006128812	0.090939897		-	—
D-Glutamine and D-glutamate metabolism	0.121478165	0.181248803	0.003034003	2.472742734	3.55E-05	T
Dilated cardiomyopathy (DCM)	0	0	1.041269841		-	—
Dioxin degradation	0.13643248	0.052507897	0.01023976	2.623572131	9.22E-05	PN
DNA repair and recombination proteins	2.013118121	3.214211944	0.002141649	3.770131405	3.55E-05	T
DNA replication	0.406516867	0.775345577	0.002520556	3.259400652	2.91E-05	T
DNA replication proteins	0.788485784	1.38382709	0.018204018	3.466035297	4.31E-05	T

Drug metabolism - cytochrome P450	0.125107436	0.042687984	0.005371678	2.621114129	0.0001332	PN
Drug metabolism - other enzymes	0.248439813	0.363001452	0.002275502	2.758779285	0.000110902	T
ECM-receptor interaction	0	0	1.138888889		-	—
Electron transfer carriers	0.098064656	0.04361345	0.002621379	2.428205919	0.000880853	PN
Endocrine and other factor-regulated calcium reabsorption	4.09E-07	2.70E-06	0.917482517		-	—
Endocytosis	0.000138328	4.28E-05	0.010922411		0.000951854	—
Energy metabolism	0.860998856	0.913723092	0.062272569	2.41554879	0.034644194	T
Epithelial cell signaling in Helicobacter pylori infection	0.070425401	0.099687992	0.003378065	2.163188533	9.22E-05	T
ErbB signaling pathway	0	0	1.009230769		-	—
Ether lipid metabolism	0.002375651	0.00201195	0.626851409		-	—
Ethylbenzene degradation	0.054785814	0.055283619	1.080496564		-	—
Fat digestion and absorption	0	0	1.082508251		-	—
Fatty acid biosynthesis	0.446467918	0.511062745	0.004748874	2.489237984	0.00032348	T
Fatty acid elongation in mitochondria	1.14E-05	4.35E-06	0.1694374		0.002291912	—
Fatty acid metabolism	0.521078579	0.250171234	0.006182497	3.127101437	0.000228141	PN
Fc epsilon RI signaling pathway	0	0	1.115646259		-	—
Fc gamma R-mediated phagocytosis	0.000138328	4.28E-05	0.004311478		0.000951854	—
Flagellar assembly	0.204606469	0.198741701	1.004542902		-	—
Flavone and flavonol biosynthesis	0.000660783	0.000960582	0.743803725		0.003005153	—
Flavonoid biosynthesis	0.005456651	0.009587285	0.011584375		0.024618761	—
Fluorobenzoate degradation	0.057919537	0.00704492	0.001985893	2.403858192	2.39E-05	PN
Focal adhesion	0	0	1.028213166		-	—
Folate biosynthesis	0.385208409	0.552403049	0.003449182	2.908034961	1.60E-05	T
Fructose and mannose metabolism	0.973870003	0.77443378	0.004551005	2.979060394	3.55E-05	PN
Function unknown	1.951148328	1.541578499	0.00255994	3.329549554	0.00032348	PN
G protein-coupled receptors	5.56E-06	0	0.004964732		0.006969947	—
Galactose metabolism	0.543106317	0.553607681	0.880773362		-	—
Gastric acid secretion	4.09E-07	2.70E-06	0.924329402		-	—
General function prediction only	3.216939412	3.605023109	0.003766349	3.285067793	0.00045517	T
Geraniol degradation	0.226687604	0.070207312	0.003681712	2.890205197	7.64E-05	PN
Germination	0.057109926	0.008897973	0.017755643	2.363774542	7.64E-05	PN
Glioma	0	0	1.01863354		-	—
Glutamatergic synapse	0.066135765	0.069521243	0.670585229		0.019427426	—

Glutathione metabolism	0.364697206	0.291530521	0.003723549	2.584032987	0.000191045	PN
Glycan bindng proteins	0	0	1.034700315		-	—
Glycan biosynthesis and metabolism	0.091815627	0.064366262	0.004147751	2.166688058	0.00074896	PN
Glycerolipid metabolism	0.394223831	0.314195815	0.002127742	2.588061657	0.000538388	PN
Glycerophospholipid metabolism	0.516676406	0.557046509	0.002849325	2.288100081	0.003012305	T
Glycine, serine and threonine metabolism	0.857040632	0.859834107	0.790633609		-	—
Glycolysis / Gluconeogenesis	1.133816338	1.037407014	0.002100464	2.677217124	0.000384083	PN
Glycosaminoglycan biosynthesis - chondroitin sulfate	3.92E-05	0.001523576	0.005285038		0.00193071	—
Glycosaminoglycan degradation	0.014992277	0.07743478	0.004615103	2.510024993	0.000538388	T
Glycosphingolipid biosynthesis - ganglio series	0.012047028	0.055992536	0.003600795	2.360139436	0.000538388	T
Glycosphingolipid biosynthesis - globo series	0.044411171	0.088736688	0.019087708	2.374972588	0.000635609	T
Glycosphingolipid biosynthesis - lacto and neolacto series	7.80E-05	9.68E-05	0.909382752		-	—
Glycosylphosphatidylinositol(GPI)-anchor biosynthesis	3.48E-07	0	0.681198082		-	—
Glycosyltransferases	0.358234333	0.472860002	0.017245912	2.738833405	0.0001332	T
Glyoxylate and dicarboxylate metabolism	0.704899211	0.426498805	0.003900861	3.137397371	2.91E-05	PN
GnRH signaling pathway	0.000138328	4.28E-05	0.002463702		0.000951854	—
GTP-binding proteins	0	0	1.096989967		-	—
Hedgehog signaling pathway	0	0	1.111864407		-	—
Hematopoietic cell lineage	5.06E-06	1.79E-06	0.148299976		-	—
Hepatitis C	0	0	1.021806854		-	—
Histidine metabolism	0.454293099	0.450874495	1.006847698		-	—
Homologous recombination	0.606111919	1.09727078	0.002229063	3.382554403	3.55E-05	T
Huntington's disease	0.065013648	0.059509999	0.625673539		-	—
Hypertrophic cardiomyopathy (HCM)	0.000415825	4.21E-05	0.026652773		0.001958268	—
Indole alkaloid biosynthesis	2.42E-05	2.38E-05	1.136320822		-	—
Influenza A	0.002104458	0.000704204	0.041341836		0.015219242	—
Inorganic ion transport and metabolism	0.403597152	0.21766742	0.006068006	2.969172273	5.23E-05	PN
Inositol phosphate metabolism	0.256140178	0.104481668	0.02047952	2.878938308	9.22E-05	PN
Insulin signaling pathway	0.058003501	0.075202867	0.016383616		0.000384083	—
Ion channels	0.029232912	0.034645391	0.173555258		-	—
Isoflavonoid biosynthesis	1.97E-05	1.71E-05	0.968560851		-	—
Isoquinoline alkaloid biosynthesis	0.066298211	0.067157994	1.003421487		-	—
Leishmaniasis	1.74E-07	3.96E-07	0.398314203		-	—

Leukocyte transendothelial migration	0	0	1.150877193		-	—
Limonene and pinene degradation	0.221401839	0.106734485	0.002952003	2.744276151	0.000635609	PN
Linoleic acid metabolism	0.02190683	0.039846966	0.005851292		0.00074896	—
Lipid biosynthesis proteins	0.610688994	0.62743954	0.064944065		-	—
Lipid metabolism	0.194631304	0.136856506	0.002753549	2.460206539	2.39E-05	PN
Lipoic acid metabolism	0.052937888	0.043748273	0.191371654		-	—
Lipopolysaccharide biosynthesis	0.328954733	0.55015651	0.003723549	3.025225718	0.00032348	T
Lipopolysaccharide biosynthesis proteins	0.565775673	0.685912726	0.014894197	2.740270628	0.001211497	T
Long-term depression	0	0	1.163120567		-	—
Long-term potentiation	0	0	1.159010601		-	—
Lysine biosynthesis	0.555055371	0.630263079	0.006934864	2.568913615	0.00045517	T
Lysine degradation	0.339742313	0.167217426	0.004681033	2.933137765	0.000538388	PN
Lysosome	0.020061245	0.081313989	0.007123311	2.50373802	0.00074896	T
MAPK signaling pathway	0	0	1.037974684		-	—
MAPK signaling pathway - yeast	0.036352277	0.039420156	0.377360345		-	—
Measles	0	0	1.054662379		-	—
Meiosis - yeast	0.002035561	0.007901219	0.002899755		0.001034009	—
Melanogenesis	0.000278012	3.99E-05	0.003640804		0.000686499	—
Membrane and intracellular structural molecules	0.791822993	0.79587129	1.035066017		-	—
Metabolism of cofactors and vitamins	0.220359115	0.148173499	0.004045337	2.560720964	0.000384083	PN
Metabolism of xenobiotics by cytochrome P450	0.123988909	0.043269201	0.002482366	2.612216595	0.0001332	PN
Methane metabolism	0.935524776	1.002060365	0.011526162	2.534839291	0.007000942	T
Mineral absorption	0.012043556	0.025368347	0.001927484		0.0001332	—
Mismatch repair	0.505290597	0.944256908	0.003810143	3.335587318	2.91E-05	T
mRNA surveillance pathway	4.74E-06	3.89E-06	1.017446322		-	—
mTOR signaling pathway	0	0	1.086092715		-	—
N-Glycan biosynthesis	0.007499948	0.036911138	0.065534466	2.175339605	7.64E-05	T
Naphthalene degradation	0.176600162	0.151707632	0.011468531	2.082645702	0.00913644	PN
Neuroactive ligand-receptor interaction	0	0	1.058064516		-	—
Neurotrophin signaling pathway	0	0	1.100671141		-	—
Nicotinate and nicotinamide metabolism	0.397181703	0.494473877	0.002155739	2.673936694	0.000384083	T
Nitrogen metabolism	0.839955263	0.736394345	0.002291415	2.70959852	0.0001332	PN
Nitrotoluene degradation	0.080771617	0.034089342	0.001950431	2.366630587	7.64E-05	PN

NOD-like receptor signaling pathway	0.020126334	0.039172031	0.003620689		0.001211497	—
Non-homologous end-joining	0.003713747	0.008703234	0.017582418		-	—
Notch signaling pathway	1.51E-06	3.05E-07	0.400049749		-	—
Novobiocin biosynthesis	0.110807411	0.11440654	0.628359875		-	—
Nucleotide excision repair	0.213884502	0.452675445	0.036408036	3.073391719	4.31E-05	T
Nucleotide metabolism	0.129152413	0.058214982	0.002391769	2.555384603	0.0001332	PN
Olfactory transduction	0	0	1.071895425		-	—
One carbon pool by folate	0.406308537	0.728475645	0.029788393	3.203251849	0.000191045	T
Oocyte meiosis	0	0	1.051282051		-	—
Other glycan degradation	0.08589691	0.195909286	0.014599262	2.760304696	0.000880853	T
Other ion-coupled transporters	1.697122832	1.3289607	0.008622956	3.265323235	2.39E-05	PN
Other transporters	0.268437614	0.216978453	0.003947859	2.41716829	3.55E-05	PN
Other types of O-glycan biosynthesis	0	0	1.012345679		-	—
Others	1.03247786	0.961095522	0.111608915	2.5380984	0.047967874	PN
Oxidative phosphorylation	0.991281612	1.171679394	0.00668719	2.965957357	0.000191045	T
p53 signaling pathway	0.002118084	0.000710047	0.041535929		0.015219242	—
Pancreatic cancer	0	0	1.078947368		-	—
Pancreatic secretion	4.09E-07	2.70E-06	0.920893233		-	—
Pantothenate and CoA biosynthesis	0.489342276	0.597125182	0.002199143	2.731577409	0.000191045	T
Parkinson's disease	0.009917795	0.017244761	0.186648794		-	—
Pathogenic Escherichia coli infection	0	0	1.015479876		-	—
Pathways in cancer	0.039180474	0.045340655	0.085922966		-	—
Penicillin and cephalosporin biosynthesis	0.029822981	0.016633755	0.003766349		0.000635609	—
Pentose and glucuronate interconversions	0.660055714	0.307538039	0.109224109	3.233436924	4.31E-05	PN
Pentose phosphate pathway	0.789187293	0.727711115	0.005285038	2.483607252	2.39E-05	PN
Peptidases	1.548708787	1.978146169	0.005201148	3.333547979	0.000191045	T
Peptidoglycan biosynthesis	0.570610205	0.998141868	0.027306027	3.323761437	2.91E-05	T
Peroxisome	0.191096598	0.184920673	0.295041059		-	—
Pertussis	0.210082331	0.036947273	0.002800618	2.934791237	1.96E-05	PN
Phagosome	0	0	1.134948097		-	—
Phenylalanine metabolism	0.370558713	0.156549276	0.004818711	2.807056874	0.000384083	T
Phenylalanine, tyrosine and tryptophan biosynthesis	0.633222384	0.764500149	0.002035232	3.021844589	1.96E-05	PN
Phenylpropanoid biosynthesis	0.138005949	0.062648581	0.004428004	2.553046933	2.91E-05	PN

Phosphatidylinositol signaling system	0.080170477	0.103472628	0.014246623	2.033180719	0.0001332	T
Phosphonate and phosphinate metabolism	0.073533634	0.047064136	0.011702583	2.134760791	0.0001332	PN
Phosphotransferase system (PTS)	1.070740985	0.388730552	0.005649523	3.529803374	0.0001332	PN
Photosynthesis	0.192392433	0.410116522	0.008401855	3.034896744	0.000191045	T
Photosynthesis - antenna proteins	0	0	1.025		-	—
Photosynthesis proteins	0.209323382	0.419980544	0.002022669	3.020190142	0.000159675	T
Phototransduction	0	0	1.123287671		-	—
Phototransduction - fly	0	0	1.154929577		-	—
Plant-pathogen interaction	0.103947125	0.129727086	0.006898365	2.134211352	0.001211497	T
Polycyclic aromatic hydrocarbon degradation	0.088056291	0.129978179	0.004255485	2.334768177	0.000538388	T
Polyketide sugar unit biosynthesis	0.088162394	0.205199831	0.003600795	2.765030318	0.001211497	T
Pores ion channels	0.658667674	0.580021172	0.009979868	2.640243254	0.002240326	PN
Porphyrin and chlorophyll metabolism	0.892304396	0.795285308	0.02047952		-	—
PPAR signaling pathway	0.107112939	0.110351675	0.729416178		-	—
Prenyltransferases	0.225434437	0.406751525	0.003561656	2.952698478	5.23E-05	T
Primary bile acid biosynthesis	0.006269609	0.002561861	0.059576787		0.001416769	—
Primary immunodeficiency	0.038114387	0.066799957	0.002501315	2.148900074	0.000110902	T
Prion diseases	0.016437617	0.006756429	0.002184482		0.000635609	—
Progesterone-mediated oocyte maturation	0.019385896	0.032148803	0.008401855		0.002600237	—
Propanoate metabolism	0.758741923	0.492883492	0.015603444	3.119099373	0.000880853	PN
Prostate cancer	0.019502342	0.032551091	0.008445163		0.002600237	—
Proteasome	0.019842509	0.033155328	0.008358988		0.001416769	—
Protein digestion and absorption	0.008119631	0.041204701	0.003343595	2.223407372	0.000538388	T
Protein export	0.411453016	0.687864642	0.002600574	3.133028997	2.91E-05	T
Protein folding and associated processing	0.740822437	0.758473102	0.238927739		-	—
Protein kinases	0.43427715	0.213727788	0.002580097	3.036251219	7.64E-05	PN
Protein processing in endoplasmic reticulum	0.035735643	0.064512531	0.005256775	2.181921757	0.001034009	T
Proximal tubule bicarbonate reclamation	0.044117108	0.016454136	0.006553447	2.135042626	7.64E-05	PN
Purine metabolism	1.758971306	2.624870757	0.006301391	3.628090814	5.23E-05	T
Pyrimidine metabolism	1.274338367	2.160790123	0.002340517	3.641426665	2.91E-05	T
Pyruvate metabolism	1.198321495	0.968016211	0.002874319	3.065740372	0.00045517	PN
Regulation of actin cytoskeleton	0	0	1.006134969		-	—
Renal cell carcinoma	0.017688435	0.012486946	0.0280862		0.00913644	—

Renin-angiotensin system	0.000677939	6.11E-05	0.003788119		0.001197624	—
Replication, recombination and repair proteins	0.602557848	0.835954459	0.00197393	3.066051815	1.30E-05	T
Restriction enzyme	0.156799555	0.226854202	0.002259809	2.547185629	9.22E-05	T
Retinol metabolism	0.076416479	0.04007604	0.002073875	2.265623019	0.00027192	PN
Rheumatoid arthritis	0	0	1.093333333		-	—
Riboflavin metabolism	0.283264545	0.32242924	0.006971752	2.273794382	0.005324894	T
Ribosome	1.429964814	2.907171308	0.004200927	3.862817258	2.91E-05	T
Ribosome Biogenesis	1.124071641	1.640520334	0.009929464	3.39658571	5.23E-05	T
Ribosome biogenesis in eukaryotes	0.038889301	0.062475847	0.003413253	2.054883311	9.22E-05	T
RIG-I-like receptor signaling pathway	0.000538479	0.001071471	0.142466229		-	—
RNA degradation	0.330215967	0.522090051	0.021844822	2.971720378	6.33E-05	T
RNA polymerase	0.102728146	0.196136397	0.081918082	2.662794356	1.96E-05	T
RNA transport	0.096744468	0.117870474	0.003744827		0.001416769	—
Salivary secretion	4.09E-07	2.70E-06	0.914096973		-	—
Secondary bile acid biosynthesis	0.004041903	0.00213473	0.597904088		0.019427426	—
Secretion system	2.072377458	1.342246258	0.005748637	3.564174249	0.000159675	PN
Selenocompound metabolism	0.377601977	0.389572256	0.066122219		-	—
Sesquiterpenoid biosynthesis	0	0	1.075409836		-	—
Shigellosis	2.20E-07	0	1.003060941		-	—
Signal transduction mechanisms	0.46840221	0.391974781	0.003062358	2.59645503	0.000538388	PN
Small cell lung cancer	0.002104632	0.0007046	0.040959041		0.015219242	—
Sphingolipid metabolism	0.074502772	0.116458887	0.104912631	2.389857297	0.001653695	T
Spliceosome	0	0	1.119453925		-	—
Sporulation	0.298951413	0.091738221	0.018995497	2.981938479	0.030971432	PN
Staphylococcus aureus infection	0.006238722	0.059934642	0.002664003	2.417101058	8.60E-06	T
Starch and sucrose metabolism	0.859142355	0.727686782	0.013106893	2.778397173	0.001926604	PN
Steroid biosynthesis	0.001531272	0.000250495	0.003702512		0.000770855	—
Steroid hormone biosynthesis	0.019484526	0.00969371	0.003681712		0.000880853	—
Stilbenoid, diarylheptanoid and gingerol biosynthesis	0.001432102	0.006059526	0.006424948		5.23E-05	—
Streptomycin biosynthesis	0.206097019	0.303397224	0.002708036	2.685902464	0.001211497	T
Styrene degradation	0.096257325	0.028713688	0.002087085	2.527143083	7.64E-05	PN
Sulfur metabolism	0.302532545	0.244129573	0.040959041	2.459821939	0.000191045	PN
Sulfur relay system	0.365293538	0.271582464	0.00511988	2.676946533	0.000635609	PN

Synthesis and degradation of ketone bodies	0.076644922	0.03805866	0.054612055	2.282864162	0.000880853	PN
Systemic lupus erythematosus	0.000681745	0.002150779	0.172899441		-	—
Taurine and hypotaurine metabolism	0.118474844	0.129399469	0.002307552		0.00032348	—
Terpenoid backbone biosynthesis	0.378638343	0.674827699	0.004488662	3.169078011	3.55E-05	T
Tetracycline biosynthesis	0.117688949	0.123388336	0.708276339		-	—
TGF-beta signaling pathway	0	0	1.064935065		-	—
Thiamine metabolism	0.390611659	0.509560422	0.003091248	2.769812782	4.31E-05	T
Tight junction	0	0	1.061488673		-	—
Toluene degradation	0.179023289	0.150144306	0.002170015	2.177405671	0.000538388	PN
Toxoplasmosis	0.002104458	0.000704204	0.04077029		0.015219242	—
Transcription factors	2.422778988	1.258439166	0.002824761	3.759679272	5.23E-05	PN
Transcription machinery	0.512465595	0.833365876	0.006826507	3.206349373	0.000384083	T
Transcription related proteins	0.034340903	0.008002146	0.002776884	2.116431143	5.22E-05	PN
Translation factors	0.348627163	0.655149901	0.00324428	3.177575827	5.23E-05	T
Translation proteins	0.744596099	1.059421874	0.009102009	3.185528374	5.23E-05	T
Transporters	8.04779164	5.502676353	0.002685839	4.097729537	0.000159675	PN
Tropane, piperidine and pyridine alkaloid biosynthesis	0.129161733	0.114700997	0.003661143		0.017210817	—
Tryptophan metabolism	0.454612322	0.181602536	0.005957679	3.133499178	0.00027192	PN
Tuberculosis	0.084280765	0.149539313	0.002323917	2.515675464	0.000384083	T
Two-component system	2.08493927	1.060246433	0.002642519	3.703164155	4.31E-05	PN
Type I diabetes mellitus	0.02717756	0.056751259	0.004890632	2.168492989	6.33E-05	T
Type II diabetes mellitus	0.03743945	0.057245221	0.002925646		2.91E-05	—
Tyrosine metabolism	0.488349877	0.362320191	0.002214002	2.788269368	0.00027192	PN
Ubiquinone and other terpenoid-quinone biosynthesis	0.393452116	0.326886445	0.00201026	2.547830886	0.00032348	PN
Ubiquitin system	0.025982169	0.013898784	0.003581118		0.00032348	—
Valine, leucine and isoleucine biosynthesis	0.580900847	0.593288408	0.731634777		-	—
Valine, leucine and isoleucine degradation	0.548332329	0.27606212	0.007447098	3.129042835	0.000538388	PN
Various types of N-glycan biosynthesis	0.000103475	0.003894819	0.012136012		0.0001332	—
Vascular smooth muscle contraction	0	0	1.104377104		-	—
Vasopressin-regulated water reabsorption	2.06E-07	3.88E-06	0.224846283		-	—
VEGF signaling pathway	1.74E-07	3.96E-07	0.399939975		-	—
Vibrio cholerae infection	0.000126548	2.74E-05	0.04809869		0.002108207	—
Vibrio cholerae pathogenic cycle	0.089738997	0.095214325	0.047879062		0.024618761	—

Viral myocarditis	0.002104458	0.000704204	0.041149548		0.015219242	—
Vitamin B6 metabolism	0.192064862	0.229157635	0.007620287	2.270635684	0.000384083	T
Wnt signaling pathway	1.51E-06	3.05E-07	0.403289019		-	—
Xylene degradation	0.089159148	0.034814387	0.002540096	2.436844755	4.31E-05	PN
Zeatin biosynthesis	0.029655768	0.07896719	0.012602782	2.390428787	0.000110902	T

*Welch's t-test corrected with Benjaminin-Hochberg FDR.

Supplementary Table S6. Predicted metagenome of the microbiota between PN and T groups from the discovery cohert us by PICRUSt. (KO and COG functional analysis in STAMP software using two-sided test with Welch's t-test corrected with Benjaminin-Hochberg FDR)

KO functional analysis related to nitrate reductase and nitrite reductase between PN and T groups from the discovery cohort

#KO	PN: mean rel. freq. (%)	PN: std. dev. (%)	T: mean rel. freq. (%)	T: std. dev. (%)	*P*-values (corrected)	Difference between means	95.0% lower CI	95.0% upper CI
K02575	0.048270943	0.01348023	0.013707193	0.013949054	1.12E-05	0.03456375	0.023263	0.045864
K00370	0.018983089	0.005151762	0.007264882	0.00459318	3.90E-05	0.011718207	0.007589	0.015848
K00374	0.024645841	0.00743199	0.007967921	0.006792014	4.65E-05	0.01667792	0.010681	0.022675
K00371	0.019036318	0.005034503	0.007233667	0.004588751	2.78E-05	0.011802651	0.007743	0.015862
K00362	0.042228353	0.01184583	0.00623527	0.010924434	1.19E-06	0.035993083	0.02641	0.045576
K00363	0.022449966	0.006296676	0.002526096	0.006035638	7.49E-07	0.01992387	0.014773	0.025075
K00368	0.003742155	0.00212624	0.009832852	0.011110525	0.071945898	-0.006090697	-0.0119	-0.00028
K03385	0.005747464	0.012844547	0.041013365	0.017591872	1.24E-05	-0.035265902	-0.04738	-0.02315

COG functional analysis related to nitrate reductase and nitrite reductase between PN and T groups from the discovery cohort

#COG	PN: mean rel. freq. (%)	PN: std. dev. (%)	T: mean rel. freq. (%)	T: std. dev. (%)	*P*-values (corrected)	Difference between means	95.0% lower CI	95.0% upper CI
COG0715	0.141626228	0.021310216	0.084443032	0.020916606	5.43E-06	0.057183196	0.039623	0.074743
COG0600	0.160322893	0.034058114	0.068887776	0.030048766	5.24E-06	0.091435116	0.064209	0.118661
COG1116	0.144063001	0.029949515	0.06741755	0.025831235	9.31E-06	0.07664545	0.052841	0.10045
COG2223	0.150026304	0.031753423	0.054283508	0.031241118	1.31E-06	0.095742796	0.069558	0.121927
COG2180	0.031012844	0.008745041	0.006866388	0.008351853	3.78E-06	0.024146456	0.017	0.031293
COG2181	0.031327344	0.00885317	0.007051523	0.008326864	4.08E-06	0.024275821	0.017074	0.031478
COG1140	0.026978011	0.007399802	0.0063404	0.006558353	3.35E-06	0.020637611	0.014715	0.02656
COG3043	0.00072584	0.000715249	0.005431305	0.004572542	0.001338408	-0.004705465	-0.00708	-0.00233
COG5013	0.026968346	0.007473441	0.008664337	0.010340293	4.98E-05	0.018304009	0.011219	0.025389
COG4459	5.10E-05	2.41E-05	5.04E-05	6.59E-05	1.064194724	6.47E-07	-0.00003	3.72E-05
COG3062	0.000496227	0.000688428	0.003722347	0.003843823	0.006406665	-0.003226119	-0.00523	-0.00122
COG3005	0.004426612	0.008174506	0.036789237	0.01424103	5.11E-07	-0.032362625	-0.04121	-0.02351

COG1251	0.055721842	0.013618722	0.016776264	0.012535758	2.50E-06	0.038945578	0.027934	0.049957
COG2116	0.060152459	0.014663793	0.037433046	0.013898223	0.001748611	0.022719413	0.010763	0.034675
COG2146	0.028114917	0.008542091	0.004588006	0.007808672	3.86E-06	0.02352691	0.016633	0.03042
COG3301	0.01380776	0.003538135	0.010175967	0.007181547	0.128052217	0.003631792	-0.00062	0.007885
COG3303	0.003910823	0.008257881	0.028346389	0.011455726	4.34E-06	-0.024435566	-0.03227	-0.01659

FDR: False Discovery Rate
