# Supplementary figures and images for: Tumor-Associated Microbiota in Esophageal Squamous Cell Carcinoma
Source: Front Cell Dev Biol. 2021 Feb 18;9:641270. doi: 10.3389/fcell.2021.641270 (PMC7930383; doi:10.3389/fcell.2021.641270)

A

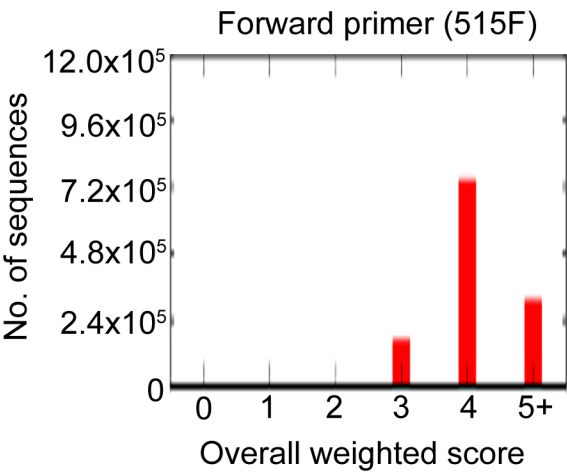

B

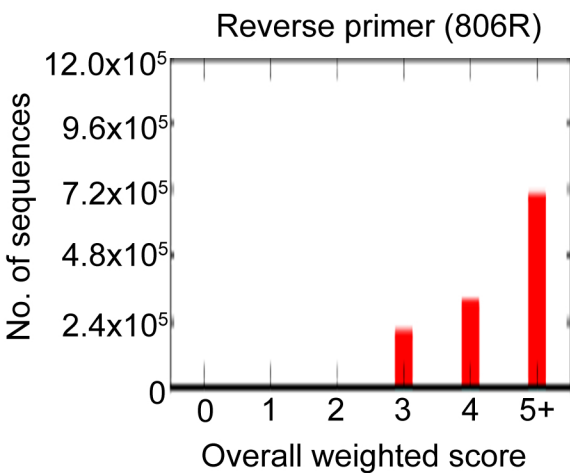

C

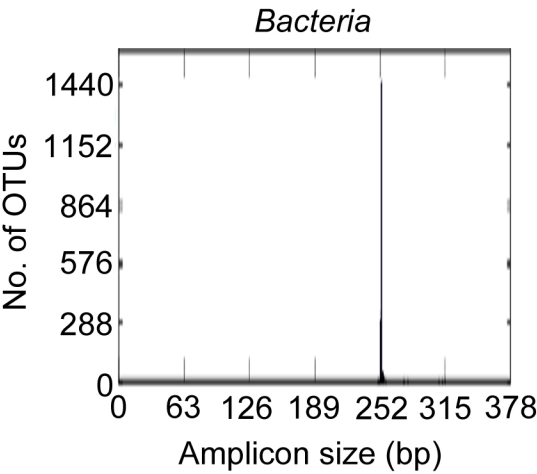

D

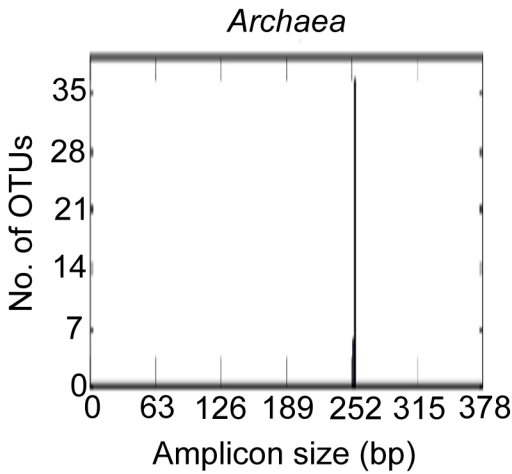

E

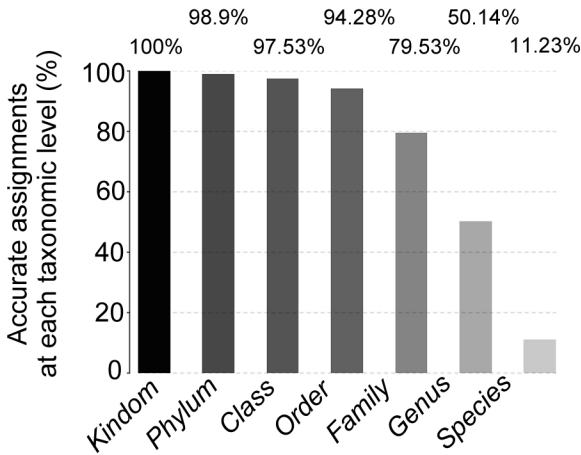

A

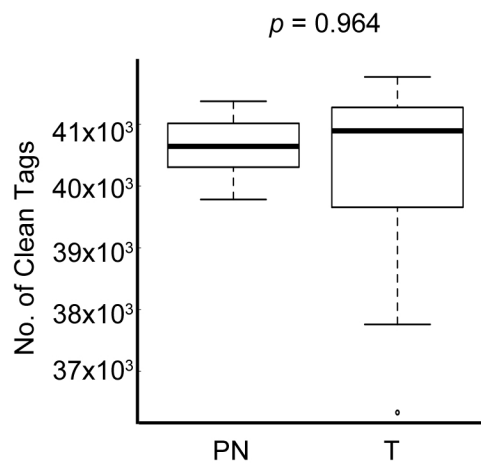

B

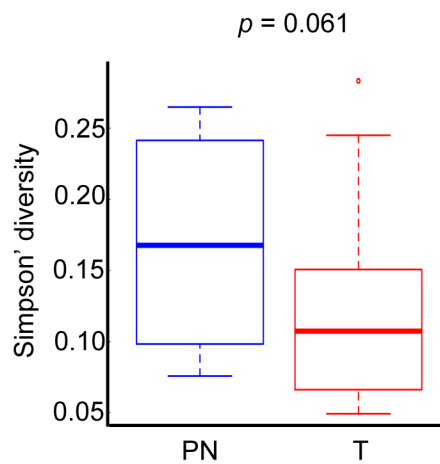

C

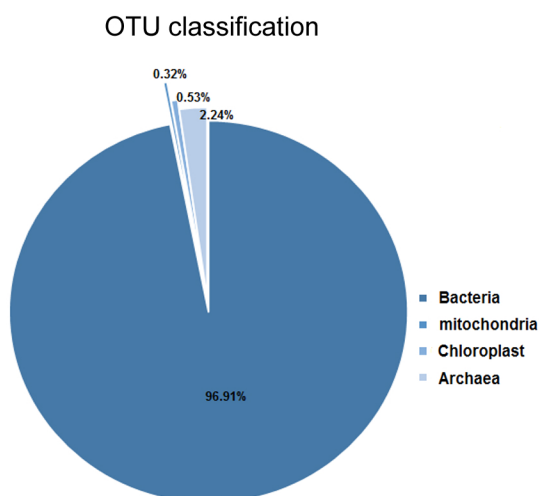

D

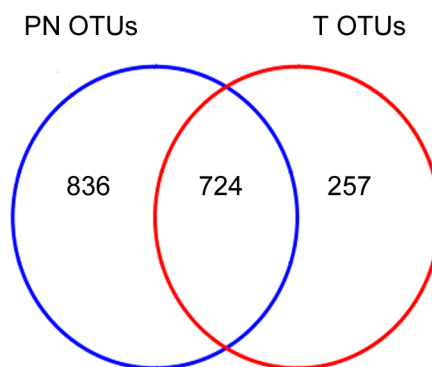

E

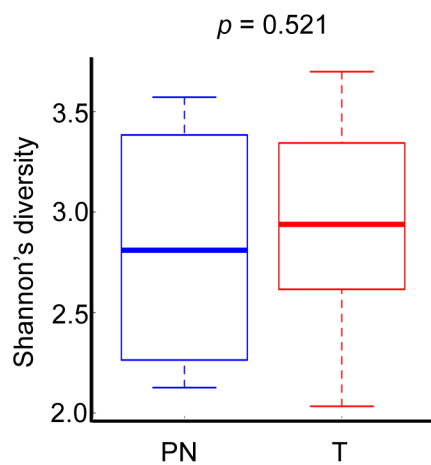

F

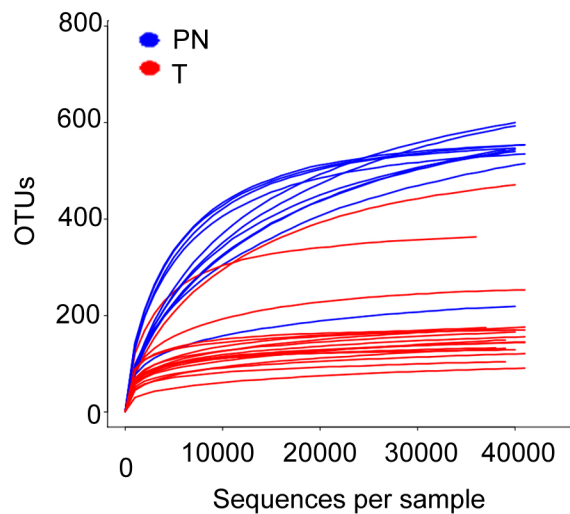

Supple Figure S2

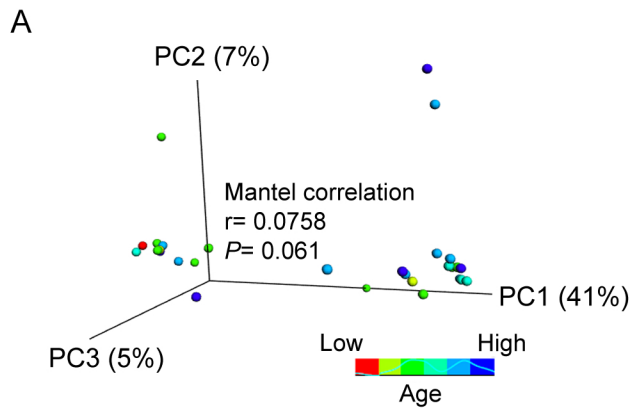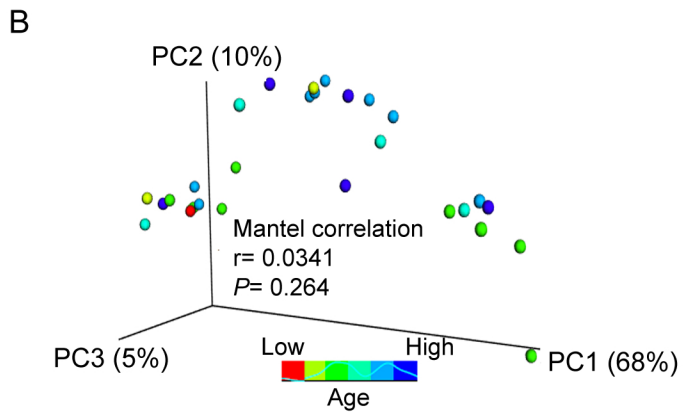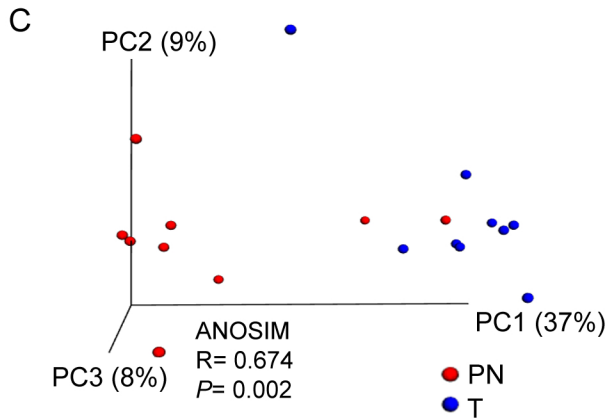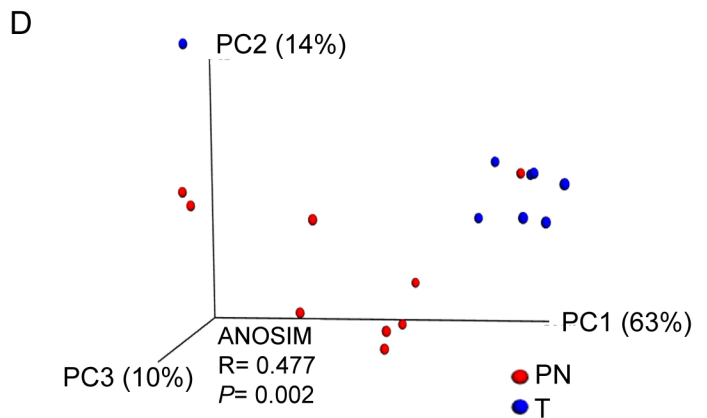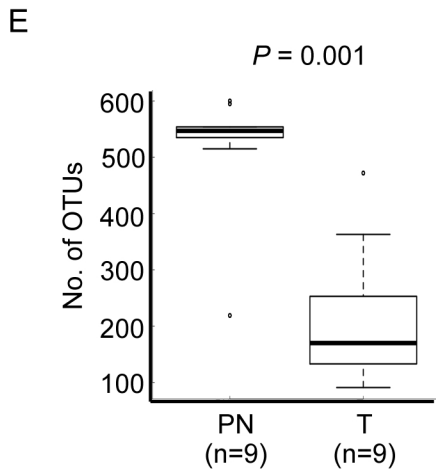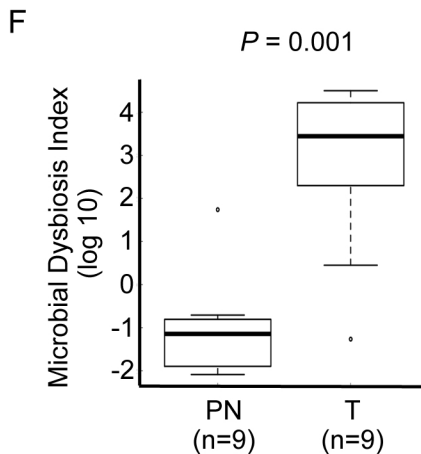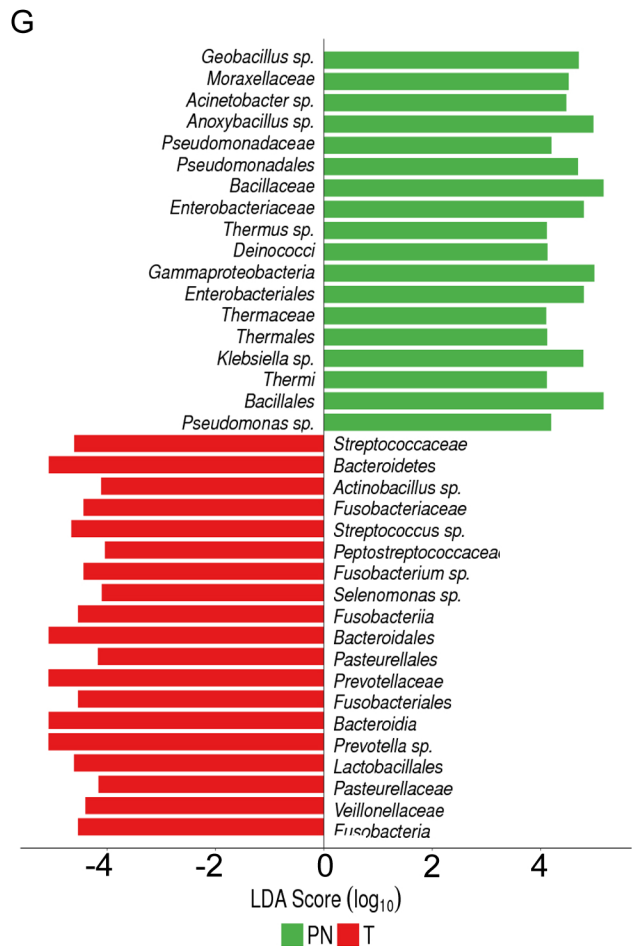

Supple Figure S3

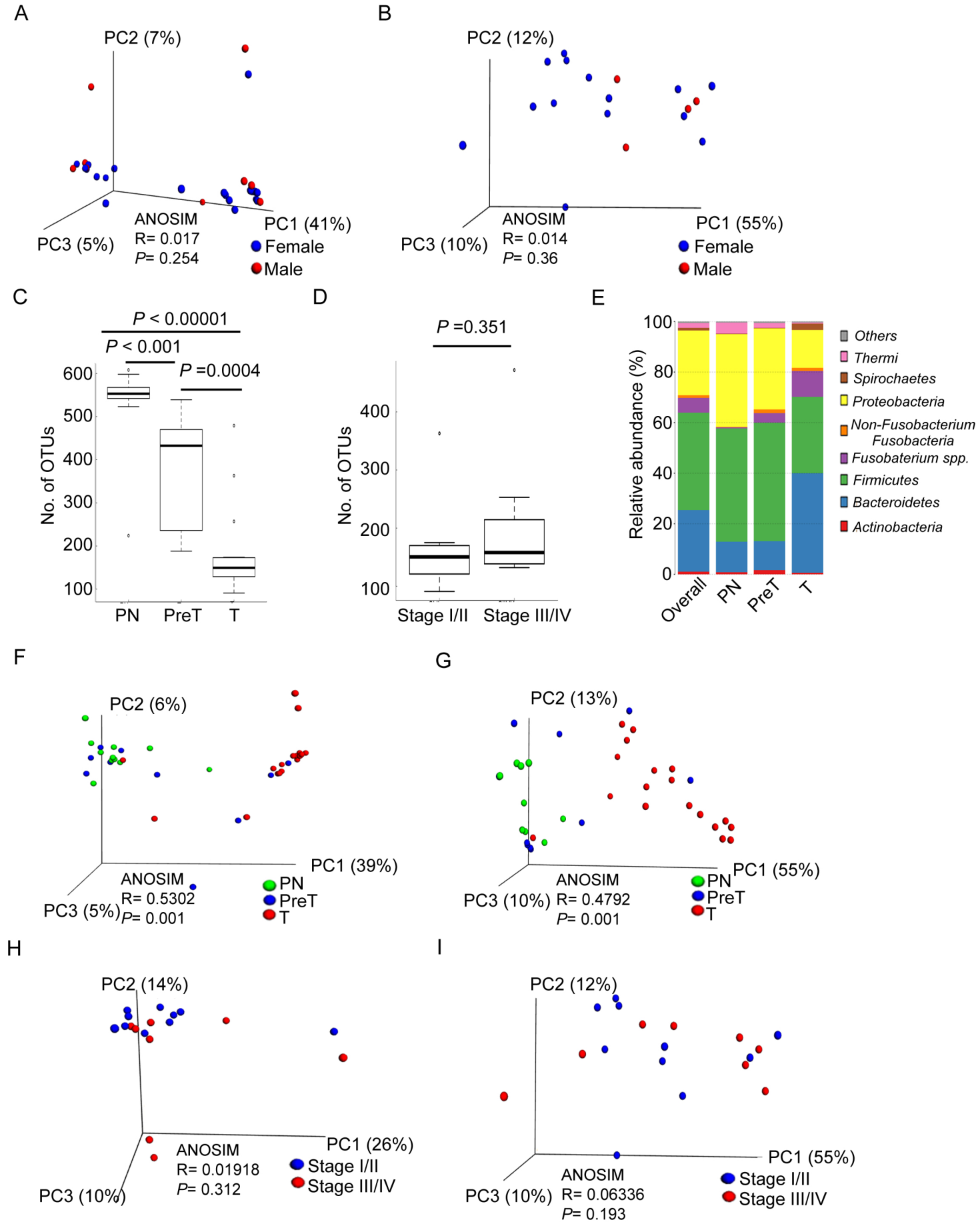

Supple Figure S4

Supplement: Supplementary Figure 1 — Performance of the primers (515F and 806R) for 16S rRNA V4 Illumina HiSeq 2500 sequencing evaluated by PrimerProspector software package. (A,B) Number of sequences matched by the forward and reverse primer. (C,D) Wrong reads matched Bacteria and Archaea. (E) Taxonomic coverage of simulated reads by taxonomic level. [file Data_Sheet_2.PDF]

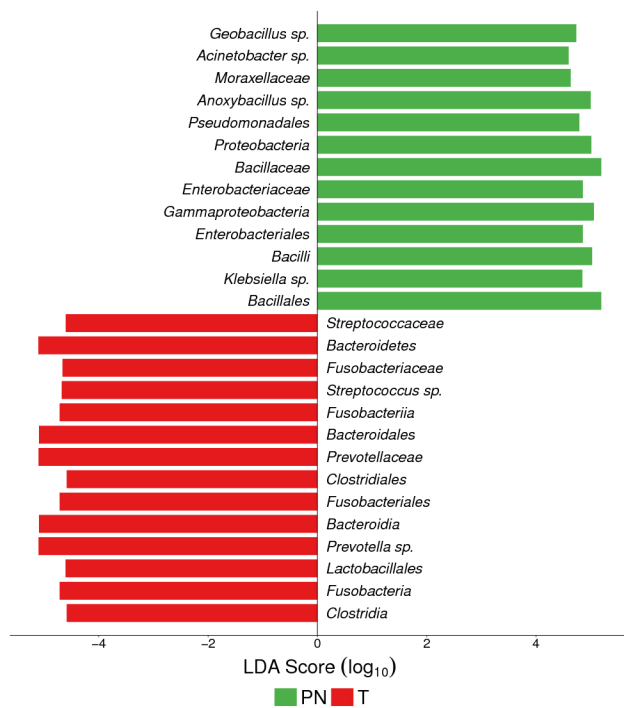

Supple Figure S5

A

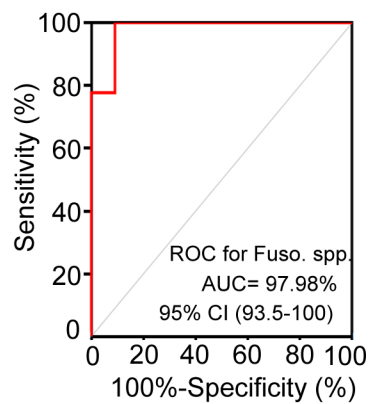

B

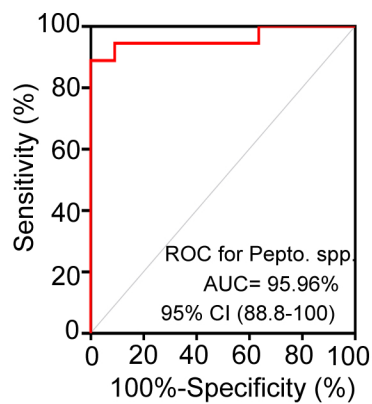

C

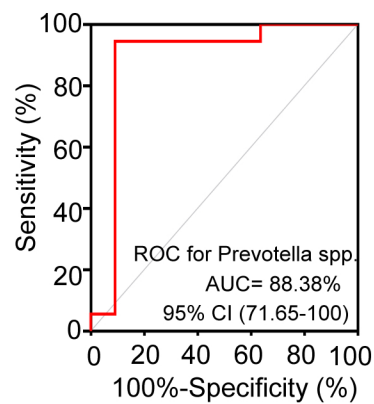

D

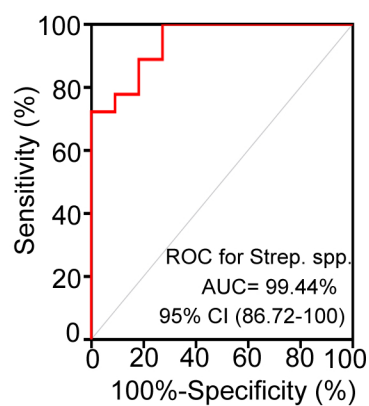

E

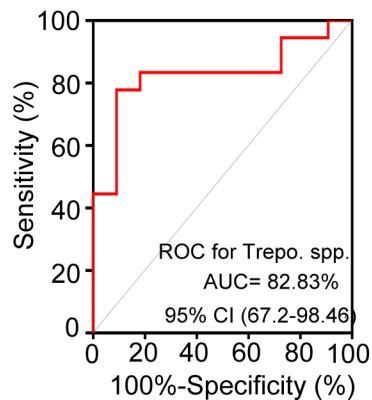

F

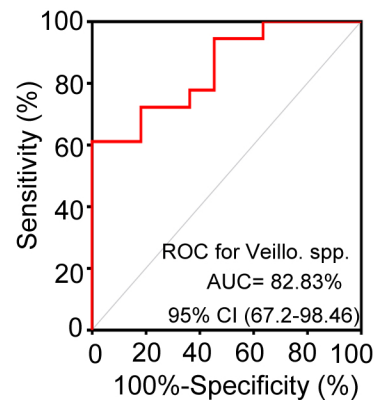

G

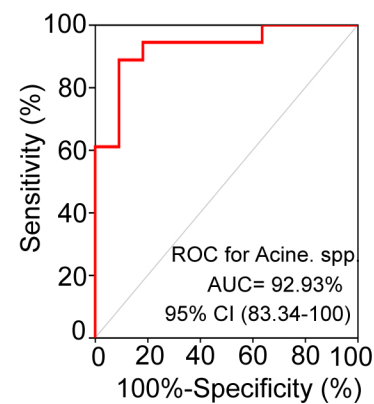

H

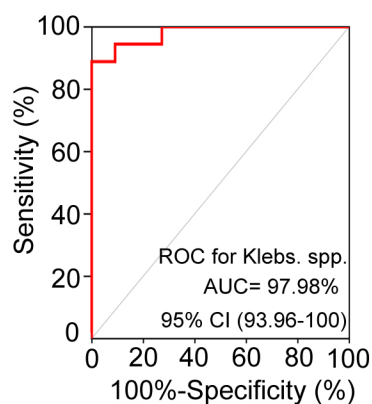

Supple Figure S6

A

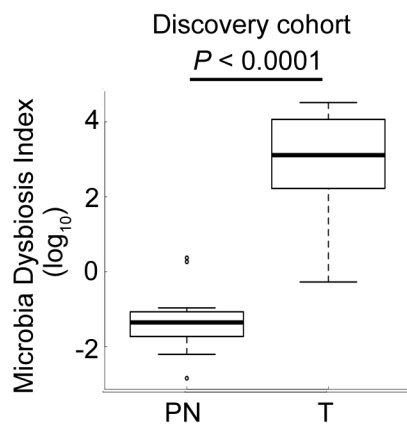

B

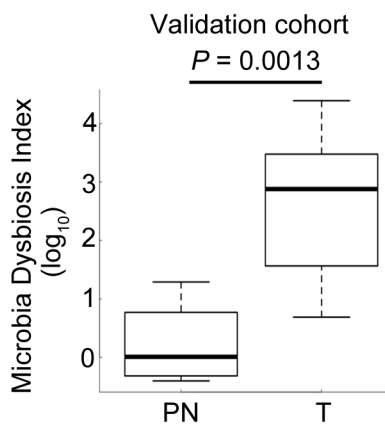

C

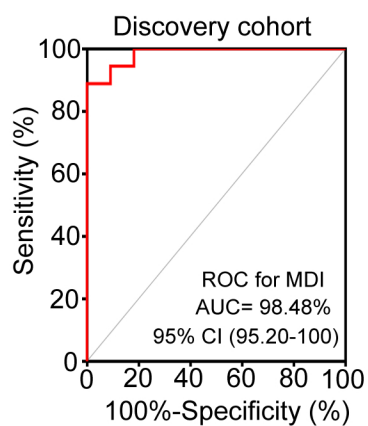

D

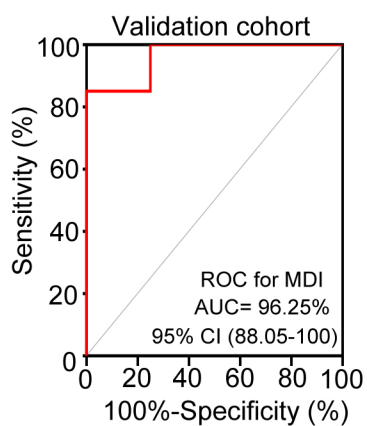

Supple Figure S7

A

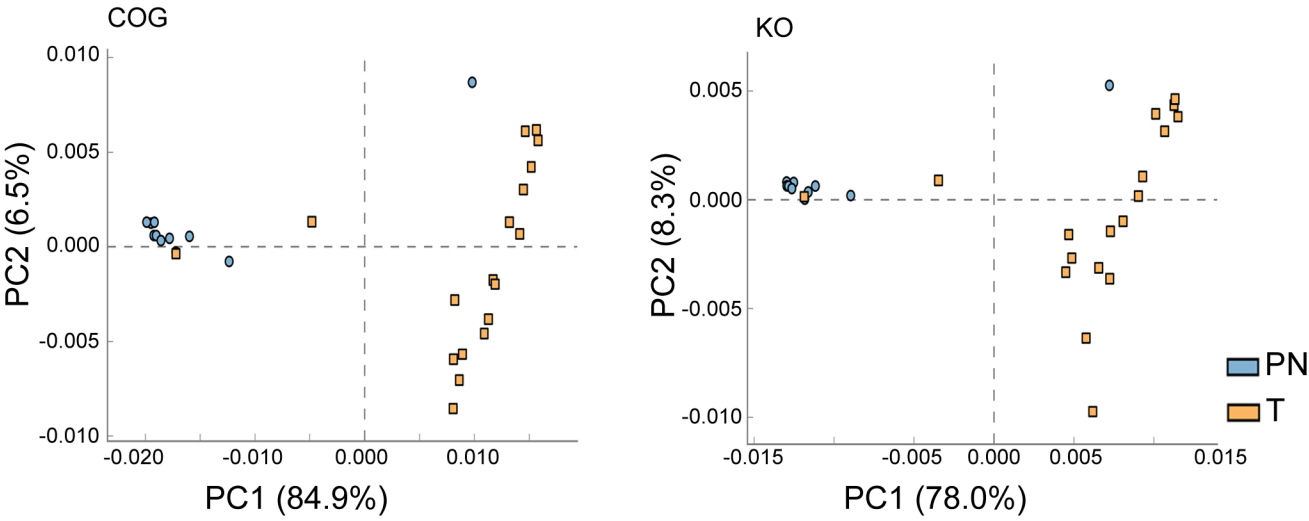

B

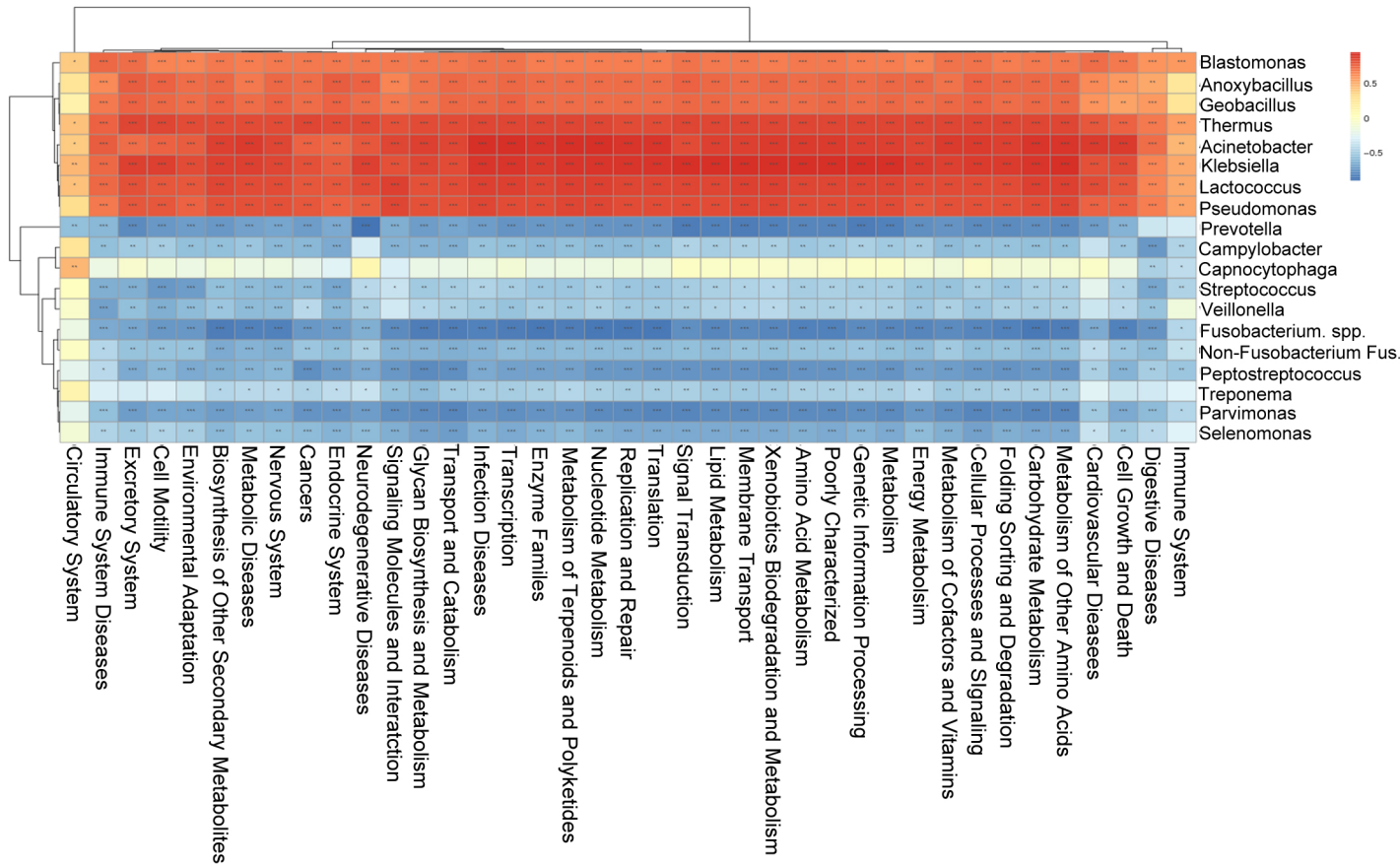

Supple Figure S8

Supplement: Supplementary Figure 5 — LEfSe reanalysis when Fusobacterium spp. was excluded from the differential results of discovery cohort. [file Data_Sheet_3.PDF]
